# Comparative analysis of phenotypic plasticity sheds light on the evolution and molecular underpinnings of locust phase polyphenism

**DOI:** 10.1038/s41598-021-91317-w

**Published:** 2021-06-07

**Authors:** Bert Foquet, Adrian A. Castellanos, Hojun Song

**Affiliations:** 1 Department of Entomology, Texas A&M University, College Station, TX USA; 2 School of Biological Sciences, Illinois State University, Campus Box 4120, Normal, IL 61790, USA; 3 Department of Wildlife and Fisheries Sciences, Texas A&M University, College Station, TX USA; 4 Cary Institute of Ecosystem Studies, Millbrook, NY USA

**Keywords:** Evolution, RNA sequencing, Non-model organisms, Behavioural methods

## Abstract

Locusts exhibit one of nature’s most spectacular examples of complex phenotypic plasticity, in which changes in density cause solitary and cryptic individuals to transform into gregarious and conspicuous locusts forming large migrating swarms. We investigated how these coordinated alternative phenotypes might have evolved by studying the Central American locust and three closely related non-swarming grasshoppers in a comparative framework. By experimentally isolating and crowding during nymphal development, we induced density-dependent phenotypic plasticity and quantified the resulting behavioural, morphological, and molecular reaction norms. All four species exhibited clear plasticity, but the individual reaction norms varied among species and showed different magnitudes. Transcriptomic responses were species-specific, but density-responsive genes were functionally similar across species. There were modules of co-expressed genes that were highly correlated with plastic reaction norms, revealing a potential molecular basis of density-dependent phenotypic plasticity. These findings collectively highlight the importance of studying multiple reaction norms from a comparative perspective.

## Introduction

Phenotypic plasticity, the ability of a genotype to produce different phenotypes in response to different environmental conditions, is ubiquitous in nature^1–4^ and is a universal property of all living things^5^. As such, predicting phenotypes from the interaction between the genetic makeup of an individual and the nature of its environment and deciphering underlying evolutionary mechanisms represent a major challenge in biology. Especially, understanding the role of phenotypic plasticity as a facilitator for evolutionary novelty and diversity is a topic that has recently received much attention^5–8^. While early studies of phenotypic plasticity mostly focused on single species that exhibit environmentally dependent alternative phenotypes (reviewed in refs.^2,3^), there is a growing recognition that a strong comparative perspective involving multiple species in a phylogenetic context is critical for evaluating the role of phenotypic plasticity in evolution^5,6,9^. By establishing ancestor–descendant relationships and quantifying reaction norms in a comparable fashion across multiple species, it is possible to infer the origin of phenotypic plasticity and subsequent modifications within a clade^5,6^. This approach has been recently proven to be powerful in a number of emerging systems, such as spadefoot toads^10^, dung beetles^11^, as well as social insects^12^.

Most of the phenotypic plasticity studies in a comparative framework have so far focused on a small number of easily quantifiable alternative phenotypes (often a single morphological trait of adaptive importance) that can be produced by manipulating environmental conditions. However, organisms often respond to different environmental stimuli in multiple ways^3^, which can involve not only morphological traits, but also behaviour as well as underlying gene expression patterns. That is to say, organisms often exhibit multiple plastic reaction norms in response to the same stimulus. However, how these individual reaction norms may differ and evolve across species is seldom addressed. Furthermore, while many plastic organisms are likely to exhibit different behaviour in response to changes in environmental conditions^3,13,14^, the evolution and mechanisms of behavioural plasticity are rarely studied. In order to understand this complex nature of phenotypic plasticity, it is critical to examine multiple plastic traits in a comparative framework to test whether these traits have followed the same or different evolutionary trajectories throughout the diversification of lineages.

One of nature’s most spectacular examples of complex phenotypic plasticity is found in locusts. Locusts are a special subset of grasshoppers (Orthoptera: Acrididae) characterised by aggregation, mass migration, and expression of an extreme form of density-dependent phenotypic plasticity known as locust phase polyphenism^15–19^. Changes in local population density cause shy ‘solitarious’ locusts that initially avoid each other, to transform into ‘gregarious’ individuals that form large migrating swarms^20^. This phenomenon influences all aspects of a locust’s life, with phenotypic changes in behaviour, colouration, morphology, reproductive and developmental biology, physiology and ecology^15–17,19–24^. Locust phase polyphenism has been intensively studied in two widespread locust species, the desert locust (*Schistocerca gregaria* Forskål) and the migratory locust (*Locusta migratoria* Linneaus)^15–17,19,20,22,24^, making it one of the best understood examples of complex and coordinated alternative phenotypes^3,19^. Nevertheless, locusts have not been prominently featured in the phenotypic plasticity literature, despite some previous attempts to place them as an exemplary system for studying the evolution of phenotypic plasticity^18^.

The grasshopper genus *Schistocerca* Stål is an ideal comparative system to study how density-dependent phenotypic plasticity has evolved across the phylogeny^25,26^ because it contains both swarming locusts and non-swarming sedentary grasshoppers. Based on the well-resolved phylogeny of the genus^26,27^, we now know that the desert locust (*S. gregaria*), for which we have extensive data on the proximate mechanisms of locust phase polyphenism^19^, occupies the basal position and is sister to the rest of the genus, which implies that the ancestral condition for the genus is the presence of density-dependent phenotypic plasticity^26^. Importantly, the three locust species in the genus, the desert locust, the Central American locust (*S. piceifrons* (Walker)), and the South American locust (*S. cancellata* (Serville)), do not form a monophyletic group, which suggests that locust phase polyphenism has been lost and gained multiple times^26,28,29^. Furthermore, recent studies have shown that non-swarming sedentary grasshopper species in the genus have the ability to exhibit varying degrees of density-dependent phenotypic plasticity in behaviour, colour, and morphology^26,30–33^, suggesting that some aspects of density-dependent phenotypic plasticity may be phylogenetically conserved^26,29,34,35^. As such, the diversification of *Schistocerca* represents a unique natural experiment on the evolution of phenotypic plasticity.

In this study, we investigate how coordinated alternative phenotypes have evolved along the phylogeny using four closely-related species of *Schistocerca* that vary in their degrees of plastic responses to changes in density as a ‘model clade’^36,37^. Representing the locust is *S. piceifrons*, a major pest that regularly swarms in Mexico and Central America^16,38–40^. Two of the non-swarming grasshoppers in our study, *S. americana* (Drury) and *S. serialis cubense* (Saussure), exhibit varying degrees of density-dependent phenotypic plasticity^30,33^. These first three species belong to the same clade^26^, are morphologically very similar to each other^41,42^, and can even hybridise in the lab and produce viable offspring^43^ (Foquet and Song, unpublished data). The fourth species is *S. nitens* (Thunberg), a sedentary grasshopper which is broadly representative of most non-swarming species in the genus^25,26,31,35,44^ and serves as an outgroup, as it belongs to a different clade than the other three species^26^ and differs morphologically^45,46^. We first measure variation in the extent of density-dependent phenotypic plasticity in each of the four species by quantifying behavioural reaction norms (activity level, attraction to conspecifics) and morphological reaction norms (nymphal colouration, size, and a morphometric ratio) after experimentally isolating and crowding the insects during nymphal development. This allows us to address whether these individual reaction norms are plastic or non-plastic in each species, and whether they are conserved, reduced, or exaggerated among the four species. We then generate species-specific and density-responsive transcriptomes and characterise molecular reaction norms (gene expression patterns) across the four species under the same rearing conditions. These data allow us to address whether the species with more distinct plasticity also have the higher amount of differentially expressed genes. They also inform us whether the differentially expressed genes are conserved among the species or uniquely present in a single species. Finally, we measure correlations between density-dependent gene expression and behavioural and morphological reaction norms. By comparing and contrasting these density-dependent reaction norms in a phylogenetic framework, we seek to understand how individual reaction norms and their molecular mechanisms have evolved along the phylogeny, how these patterns have ultimately led to the evolution of locust phase polyphenism, and how our insights can advance our understanding on the role of phenotypic plasticity in evolution in general.

## Results

### Locust and non-swarming grasshopper species exhibit varying degrees of density-dependent behavioural plasticity

Density-dependent behavioural plasticity has been thoroughly characterised in *S. gregaria*^47^, which provided a baseline for studying our four *Schistocerca* species (hereinafter referred to by their specific epithet, *piceifrons*, *americana*, *cubense*, and *nitens*). In *S. gregaria*, crowd-reared nymphs are typically attracted towards conspecifics and are highly active, whereas those reared in isolation walk less and avoid other locusts^47,48^. Therefore, we predicted that *piceifrons*, a known locust for which behaviour had never been quantified in a standardised manner, would show distinct and characteristic behavioural plasticity similar to that of *S. gregaria*, while the behavioural plasticity in the non-swarming species would be reduced (*americana* and *cubense*) or even absent (*nitens*).

**Figure 1:**
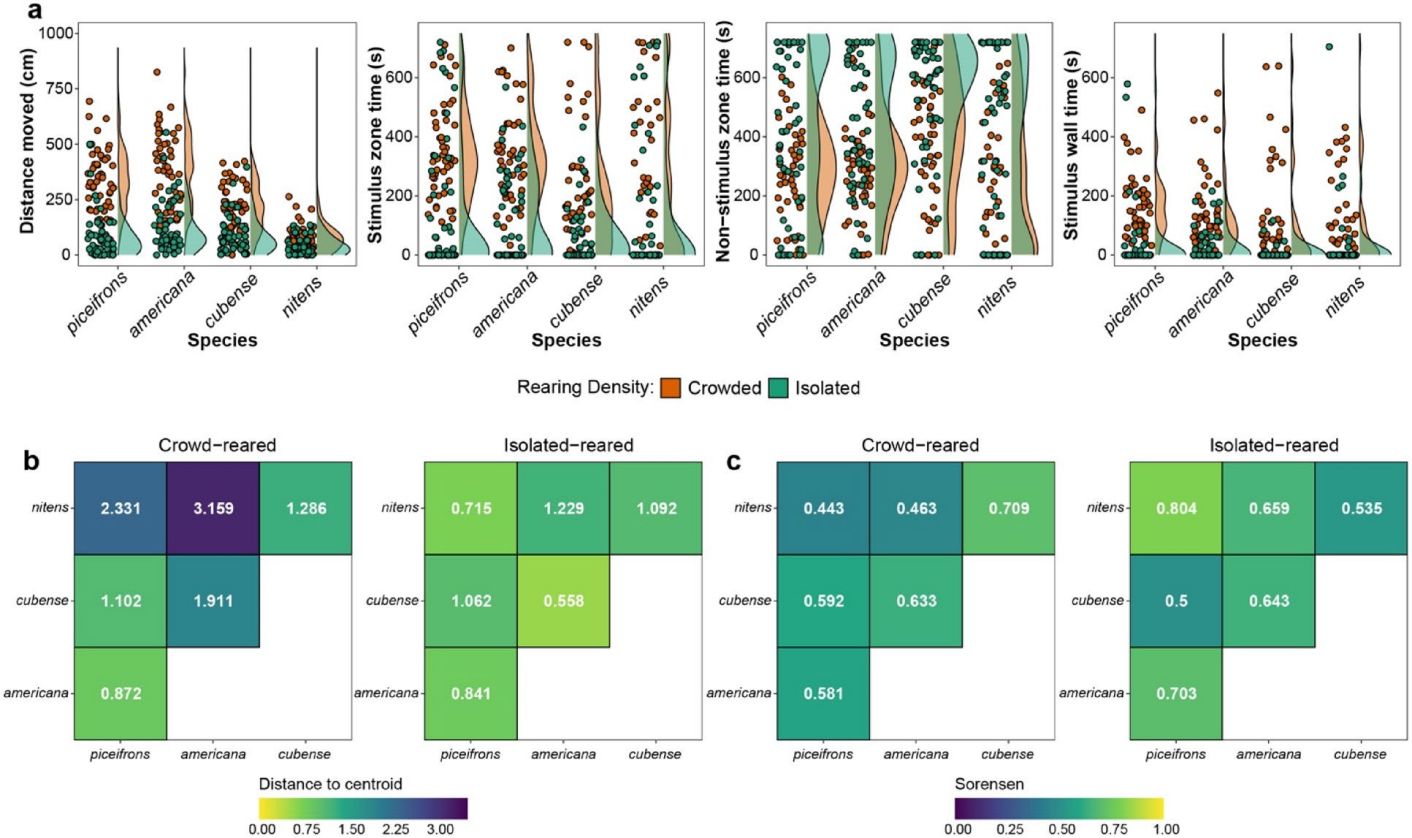
The behaviour of locust and non-swarming grasshopper species is influenced by rearing density, but varies among species. **(a)** Raincloud plots for four relevant behavioural variables in crowd-reared and isolated-reared nymphs of *piceifrons, americana, cubense* and *nitens*. The activity-related behaviour is ‘Distance moved’, in cm. The attraction-related behaviours are represented by three variables: ‘Stimulus zone time’, ‘Non-stimulus zone time’, and ‘Stimulus wall time’, all in seconds (s). Density curves show the general patterns for each rearing condition, while jittered points detail the observed data. (**b**) Heatmaps showing the distance to centroid for pairwise species comparisons of crowd-reared (left) and isolated-reared (right) nymphs. After a reduction of the behavioural data with a PCA, hypervolumes were generated for each combination of species and rearing density. Distance to centroid uses Euclidean distances to measure how different hypervolumes are. Shorter distances are shown with warmer colours approaching yellow, while larger distances are displayed as cooler colours approaching purple. **(c)** Heatmaps created using the same data and organization as **(b)** but visualizing overall similarity of each group with the Sorensen index. As before, warmer colours represent higher similarity and cooler colours represent less similarity.

We characterised behavioural responses of test insects using an assay system originally developed for *S. gregaria*, in which the behaviour of a single individual was recorded in a rectangular arena with a chamber containing crowd-reared conspecifics on one end, and an identical but empty chamber on the other end^47–50^. We measured ten behavioural variables (5 activity-related and 5 attraction-related) from crowd-reared and isolated-reared nymphs of the four *Schistocerca* species (Fig. 1a, Supplementary Fig. S1). Using a nonparametric multivariate analysis of behavioural traits, we found a significant interaction effect between species and rearing density (Supplementary Table S1), indicating that the four species differed in the magnitude of behavioural reaction norms. We subsequently compared each variable separately across species and rearing densities with a two-step hurdle model, involving logistic regression of zero and nonzero values and modeling of nonzero data^31^ (Supplementary Fig. S2, Tables S2, S3). This analysis showed that, overall, crowd-reared individuals were more active, and spent more time close to the stimulus and less time away from the stimulus than isolated-reared individuals (Fig. 1a, Supplementary Fig. S2, Tables S2, S3). This was particularly the case for *piceifrons, americana,* and *cubense*, but even *nitens* exhibited density-dependent differences for most activity-related variables and for one attraction-related variable (Fig. 1a, Supplementary Figs. S1, S2, Supplementary Tables S2, S3). When comparing the overall similarity in behaviour of all four species using a hypervolume analysis (Fig. 1b,c), we found that *piceifrons* and *americana* behaved very similarly when crowded, and somewhat similarly to *cubense*, in which the behavioural reaction norms were generally less pronounced (Fig. 1a,b). Not surprisingly, *nitens* appeared to be very different from the other three species (Fig. 1b). In contrast, the isolated-reared nymphs of all four species behaved much more similarly to each other (Fig. 1c)*.* As a result, *piceifrons* and *americana* were seemingly indiscernible from each other (Fig. 1). In summary, density-dependent reaction norms in behaviour were most plastic in *piceifrons* and *americana*, followed by *cubense*, and the least plastic in *nitens*, which was partially consistent with our predictions.

### Rearing density influenced nymphal colouration, size, and morphometric ratios of locust and non-swarming grasshoppers

Among locust species, solitarious nymphs are often cryptically coloured and green, while gregarious nymphs exhibit black patterns on a yellow, orange, or red background colouration^15,16,29,38,40,51–53^. In addition, solitarious and gregarious locusts exhibit species-specific differences in their size^20,28,51^, and the ratio of the hind femur length over the head width (F/C ratio) is considered a reliable predictor of the phase state, with larger values observed in solitarious individuals^20,40,51,54^. Thus, we hypothesised that the four species would exhibit varying levels of plastic reaction norms in these traits, and that there would be a similar spectrum of reaction norms in morphology among the species. Specifically, we expected that *piceifrons* and *americana* would exhibit the most plastic reaction norms in morphology, while the reaction norms of *cubense* and *nitens* would be less plastic.

**Figure 2:**
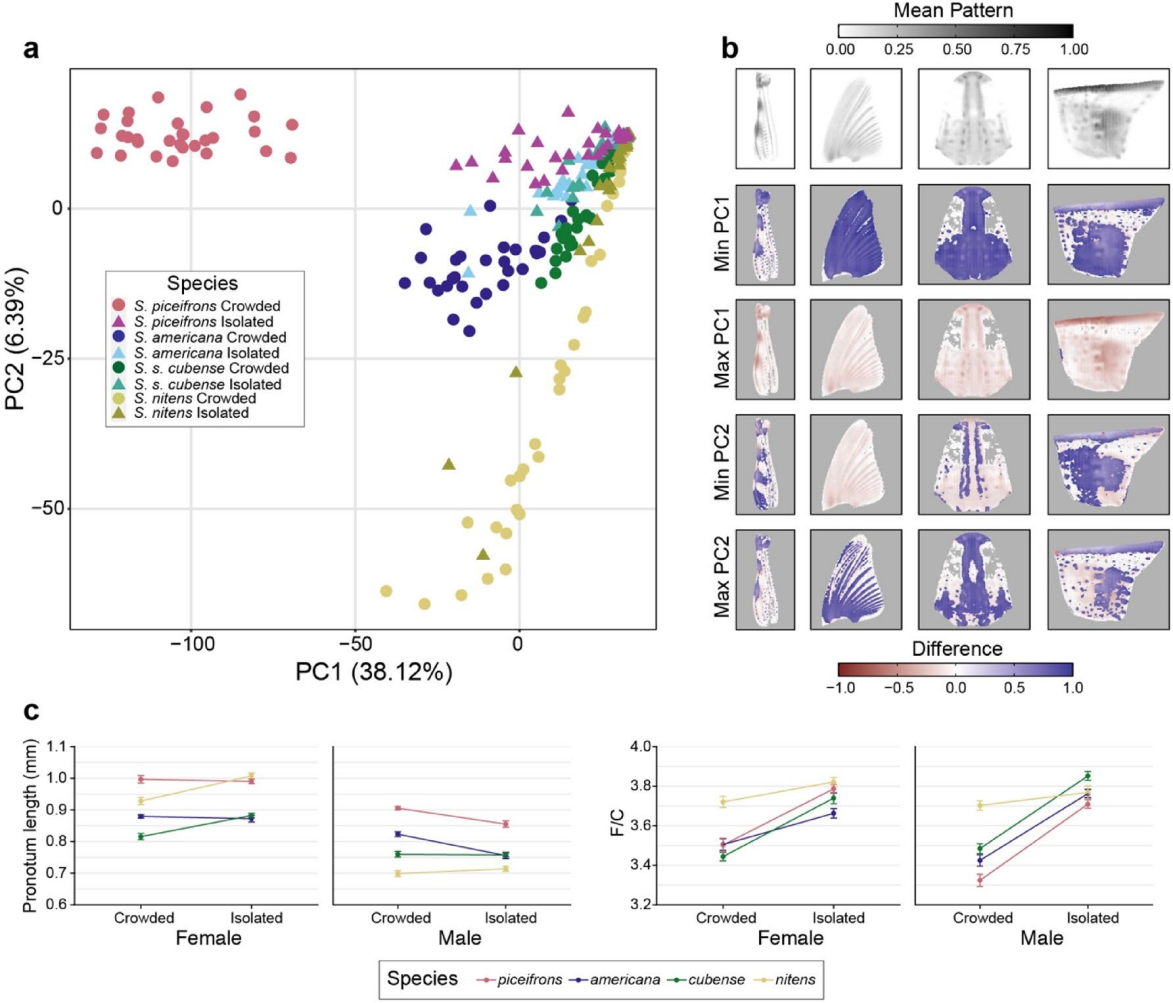
Locust and non-swarming grasshopper species all exhibit morphological responses to rearing density, but they vary among species. (**a**) Plot showing the results of a PCA analysis of the black patterns in four different tissues (hind femur, wing pad, frontal side of the head, and lateral side of the pronotum) for crowd-reared and isolated-reared nymphs of *piceifrons*, *americana*, *cubense* and *nitens*. Black patterns were detected for each body region separately using the patternize package in R^55^ and were converted to specimen- and body region-specific matrices of pixel coordinates associated with a binary depiction of the presence or absence of black colour, and the variance–covariance matrix obtained from these binary matrices was used for a PCA. (**b**) Plots showing the differences in black patterns compared to the average, associated with the minimum and maximum values of each principal component. The first row shows the average black pattern for each measured body region (hind femur, wing pad, frontal side of the head, lateral side of the pronotum), with darker patterns representing spots where a high proportion of individuals had a black pattern. Plots on the lower rows show the difference in the black pattern between this average, and the maximal or minimal value of either PC1 or PC2, with bright blue signifying patterns that are not often found in the average, and bright red indicating the absence of pattern usually found in the average. (**c**) Plots showing the mean pronotum length and F/C ratio for females and males of crowd-reared and isolated-reared specimens of *piceifrons, americana, cubense*, and *nitens*. Error bars show the standard error of mean.

Using the R-package patternize^55^, we extracted the black patterns from four different body regions for each species and rearing condition, and quantified their variation with a principal component analysis (Fig. 2a,b). All four species exhibited differences in the extent of the black patterns between isolated-reared and crowd-reared individuals (Fig. 2a), but they were by far the largest for *piceifrons*. This species exhibited a clear separation of crowd-reared nymphs and isolated-reared nymphs over the first principal component, with crowd-reared nymphs exhibiting clear black patterns on all four investigated body regions and isolated-reared nymphs generally showing only small black dots in combination with a narrow black line running longitudinally over the thorax (Fig. 2a,b). Crowd-reared nymphs of *americana* and to an even larger extent *cubense* exhibited less extensive black patterns than their *piceifrons* counterparts, while isolated-reared nymphs of all three species were highly similar. For both *americana* and *cubense*, there was an overlap between crowd-reared and isolated-reared nymphs, and they could only partially be separated, even when combining the first and the second principal component (Fig. 2a). In *nitens*, the black patterns, if present at all, had a different shape and location than in the other three species (Fig. 2a,b). Although crowd-reared nymphs of *nitens* exhibited an overall larger extent of black patterns than isolated-reared nymphs, there was a large overlap between both rearing conditions. In summary, density-dependent reaction norms in nymphal black patterns were the most plastic in *piceifrons*, less plastic in *americana*, and the least plastic in *cubense* and *nitens*.

We also measured pronotum length as a proxy for grasshopper size^56^ and calculated the F/C ratio for each specimen. We tested the effect of sex, species, and rearing condition on the pronotum length with a three-way ANOVA (Fig. 2c, Supplementary Table S4), and found significant two-way interactions for each possible combination, but no significant three-way interaction (Supplementary Table S4). This suggested that the rearing condition differentially influenced grasshopper size in different species and in different sexes. The pronota of isolated-reared males of *piceifrons* and *americana* were significantly smaller than crowd-reared males of the same species (respectively *p* = 0.009 and *p* < 0.001, Tukey-adjusted post-hoc test), but no significant differences were observed for females of these two species (Fig. 2b). In contrast, the pronota of isolated-reared females of *nitens* and *cubense* were significantly larger than their crowd-reared counterparts (*p* < 0.001 for both comparisons, Tukey-adjusted post-hoc test), but surprisingly no significant differences were observed for male specimens of these species (Fig. 2b). For the F/C ratio, we found a significant three-way interaction between sex, species and rearing-condition (Supplementary Table S5), and the F/C ratios were significantly higher in isolated-reared nymphs of all species except *nitens* (*p* = 0.004 for female *americana, p* < 0.001 for all other comparisons, Tukey-adjusted post-hoc test) (Fig. 2c). In other words, density-dependent reaction norms of the morphometric ratio were similarly plastic in *piceifrons*, *americana* and *cubense*, and the least plastic in *nitens*, while the reaction norms for nymphal size were similarly plastic in *piceifrons* and *americana*, but exhibited a different pattern in *cubense* and *nitens*.

### Locust and grasshopper species differed in the number of density-responsive genes, consistent with variation in plasticity expression

After quantifying density-dependent reaction norms in behaviour and morphology in the four *Schistocerca* species, we sought to characterise the transcriptomic response to rearing density. Previous transcriptomic studies in *S. gregaria*^57,58^ and *L. migratoria*^59–67^ showed hundreds of density-responsive genes, involved mostly in metabolism but also several other processes, that were differentially expressed between solitarious and gregarious individuals. We therefore expected that there would be an equally large number of density-responsive genes in the locust *piceifrons*, involved in similar molecular processes. Additionally, we expected that the number of density-responsive genes for each species would be correlated to the extent of its reaction norms in behaviour and morphology.

**Figure 3:**
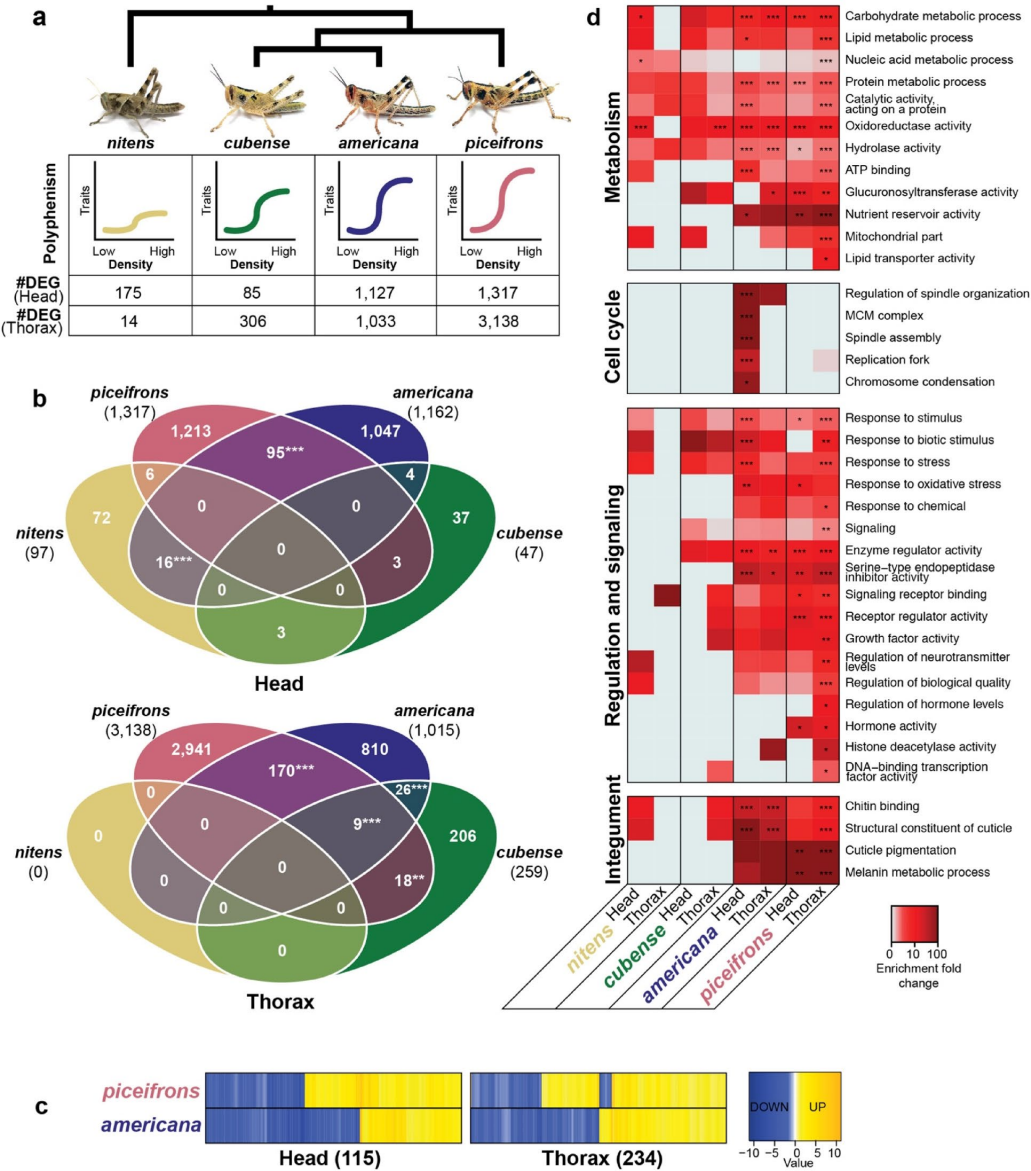
Behaviourally similar species do not exhibit a large overlap in density-responsive genes, but the molecular response to density is functionally conserved. (**a**) Short overview of the phylogenetic relationships between the four studied species^26^, the relative extent of polyphenism as expressed in reaction norms, and the number of density-responsive genes for the head and the thorax tissue of each species. The number of density-responsive genes represents the amount of differentially expressed genes found by either DEseq2 or edgeR, using the species-specific transcriptomes as reference. The pictures show representative crowd-reared individuals from our lab colonies. (**b**) A dendrogram depicting the overlap of density-responsive genes between the four studied species for either the head or the thorax tissue. Each number represents the number of density-responsive genes found by either edgeR or DEseq2, with the *piceifrons* transcriptome as reference. The numbers listed under the species epithet represent the total number of density-responsive genes for that species, while all other numbers represent genes exhibiting similar expression patterns in one or more species. Significance levels of overlap were obtained with a Super Fisher exact test (*p < 0.05, **p < 0.01, ***p < 0.001). (**c**) Heatmaps showing all genes that were differentially expressed between isolated-reared and crowd-reared nymphs in both *piceifrons* and *americana* for the head tissue and the thorax tissue. The isolated-reared condition was used as reference, and for each gene, the highest log_2_foldchange of edgeR or DEseq2 was used. Blue colours represent genes that are downregulated in crowd-reared individuals while yellow colours represent genes upregulated under crowd-reared conditions. While most density-responsive genes show a similar expression pattern in both species, the expression pattern is reversed between both species for some genes. (**d**) Heatmap showing enrichment of a representative set of GO terms for density-responsive genes in all species-tissue combinations. GO terms were arbitrarily divided in four different groups. Darker colours indicate a higher fold change of enrichment in the density-responsive genes compared to the whole transcriptome. Significance was calculated with a Fisher exact test in Blast2GO, with as reference the transcriptome for the tested tissue and species. *: FDR < 0.05, **: FDR < 0.01, ***: FDR < 0.001.

We generated species-specific transcriptomes for head and thorax tissues from the four species, and found that they had a similar quality and composition (Supplementary Tables S6–S10) and contained between 278,171 and 374,276 contigs (Supplementary Table S6). We first aligned all reads to their representative transcriptomes and used edgeR^68^ and DEseq2^69^ to find density-responsive genes (Supplementary Data Files 1–8). As expected, we found that *piceifrons* had the highest number of density-responsive genes among the four species, with 1,317 density-responsive genes in the head tissue and 3,138 density-responsive genes in the thorax tissue (Fig. 3a). Although *piceifrons* and *americana* exhibited similar reaction norms in both behaviour and morphology, the number of density-responsive genes in *americana* was slightly lower in the head tissue (1,127) and much lower in the thorax tissue (1033) (Fig. 3a). *Cubense* and *nitens*, which showed less plastic reaction norms compared to *piceifrons* and *americana*, had much lower numbers of density-responsive genes (Fig. 3a). These results suggested that there is indeed a spectrum of plastic reaction norms at the transcriptomic level among these four species.

### Density-responsive genes were largely species-specific, but functionally conserved

Given that the locust *piceifrons* exhibited the most extensive plastic reaction norms overall, we posited that the set of density-responsive genes for each non-swarming species would be a subset of those found in *piceifrons.* To assess the overlap of density-responsive genes among different species, raw reads for each species were aligned to either the head transcriptome or the thorax transcriptome of *piceifrons* (Fig. 3b, Supplementary Table S11). The observed overlap between the density-responsive genes in different species was relatively low, although statistically significant for several comparisons (Fig. 3b, Supplementary Figs. S3, S4, Supplementary Data Files 9,10). Surprisingly, we did not find a single density-responsive gene that was shared by all four species in either tissue (Fig. 3b). Among *piceifrons*, *americana*, and *cubense*, which belong to the same clade^26^, we found only nine shared density-responsive genes for the thorax tissue and none for the head tissue (Fig. 3b, Supplementary Data Files 9,10). Similarly, between *piceifrons* and *americana*, only 95 density-responsive genes were shared in the head tissue and 170 additional genes were shared in the thorax tissue, or respectively 8.2% and 17.6% of all density-responsive genes of *americana* (Fig. 3b,c). In addition to these density-responsive genes with similar expression patterns in *piceifrons* and *americana*, there were respectively 26 and 69 genes for head and thorax tissue that were density-responsive for both species, but that showed an opposite response (Fig. 3c, Supplementary Data Files 9,10). These surprising observations indicate that the patterns of differential expression are often species-specific, rather than conserved across species.

We subsequently sought to investigate whether the transcriptomic responses to density in different species were more similar to each other at a functional level^70^. Using Fisher exact tests, we showed that to a large degree, the same gene ontology (GO) terms were enriched among density-responsive genes in different species and tissues (Fig. 3d, Supplementary Data File 11). In both *piceifrons* and *americana*, the set of density-responsive genes showed an enrichment of many GO terms involved in metabolism, signaling, and integument (Fig. 3d, Supplementary Data File 11). All major branches of the cellular metabolism were found to be enriched, such as lipid, carbohydrate, protein and nucleic acid metabolism. Additionally, there was a large variety of processes related to signaling that were enriched, such as hormone and neurotransmitter production, growth factor activity, histone modifications, and the cellular responses to a large number of stimuli. Finally, there was a strong enrichment of GO terms involved in the integument, including cuticle pigmentation and the melanin metabolic process, potentially involved in the density-dependent nymphal colouration. The overall number of enriched GO terms was lower in *americana* than in *piceifrons*, and they mostly represented a subset of those found in *piceifrons* (Fig. 3d, Supplementary Data File 11). Nonetheless, several GO terms were only enriched in *americana* (Supplementary Data File 11), in particular one large group of GO terms involved in the cell cycle (Fig. 3d). Compared to *piceifrons* and *americana*, the number of enriched GO terms found in *cubense* and *nitens* was much smaller (Supplementary Data File 11), most likely a result of their much lower numbers of density-responsive genes (Fig. 3a). Still, most enriched GO terms found in *cubense* and *nitens* were a subset of the GO terms shared between *piceifrons* and *americana*, and were involved in metabolism (Fig. 3d, Supplementary Data File 11).

These results collectively demonstrate that density-responsive genes are functionally similar across species, although the response of an individual gene to different rearing densities is generally species-specific. This suggests that different species have independently evolved different, but functionally similar, patterns of density-responsive gene expression.

### A potential role for hexamerins in locust behavioral polyphenism

Finally, we sought to better understand the molecular underpinnings of the different phenotypically plastic traits. We used Weighted Gene Co-expression Network Analysis (WGCNA) to cluster genes with similar expression patterns into modules. We then took advantage of the quantification of the reaction norms of behaviour and morphology (Figs. 1,2) to evaluate whether gene expression in these modules could be correlated with these reaction norms^71^.

**Figure 4:**
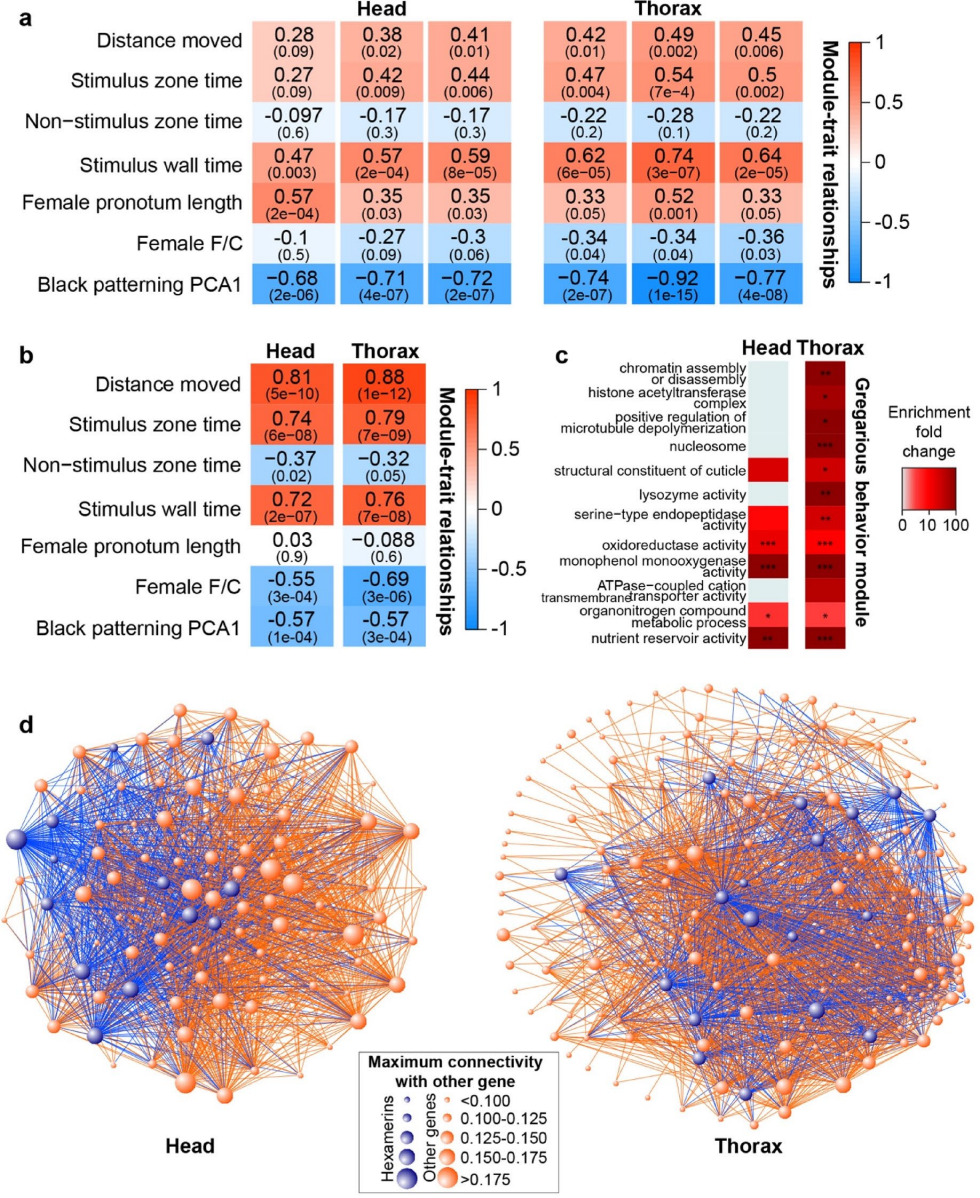
Groups of co-expressed genes exhibit high correlation with phenotypic traits. **(a,b)** Heat plots depicting correlations of different phenotypic traits and gene co-expression modules that are highly correlated with (**a**) nymphal colouration or (**b**) behaviour. Phenotypic trait values for sequenced values were inferred from the measured individuals. Stronger correlations are shown by more intense colours, with red signifying a positive correlation and blue a negative correlation. The upper number in each square represents the correlation value between the eigenvalue of the module and the behavioral trait, while the lower value refers to the associated *p* value, both calculated by WGCNA. (**c**) Heatmap showing enrichment of GO terms in the two gregarious behaviour modules with the respective *piceifrons* transcriptome as reference. GO terms were arbitrarily divided in four different groups. Darker colours refer to a higher fold change of enrichment in the density-responsive genes compared to the whole transcriptome. Significance was calculated with a Fisher exact test in Blast2GO, with as reference the transcriptome for the tested tissue and species (*p < 0.05, **p < 0.01, ***p < 0.001). (**d**) Network graphs, made with VisANT v5.51, showing the connections between all genes included in the gregarious behavior module for the head tissue and for the thorax tissue. Larger dots represent genes with a higher connectivity. Blue dots represent hexamerin-like sequences and partial hexamerin-like sequences, which were generally found to have high connectivities. All other genes are coloured orange.

Using the raw reads of all four species, aligned to their respective *piceifrons* transcriptome, we found 36 and 45 modules of connected genes for the head tissue and the thorax tissue, respectively (Supplementary Figs. S5, S6, Supplementary Data Files 12,13). Six of these modules (three in the thorax and three in the head) showed very strong negative correlations (< -0.7) with the first principal component of our analysis of the black pattern (Fig. 4a), or in other words a strong positive correlation with the extent of black patterning (Fig. 2a). These modules contained mostly metabolic genes, in addition to cuticular proteins and potential regulatory genes, such as pacifastins and transcription factors (Supplementary Data Files 12,13).

We also identified one module in each tissue that exhibited very high correlations with multiple behavioural variables (e.g. correlations of 0.81 and 0.88 with ‘distance moved’ for head tissue and thorax tissue, respectively) (Fig. 4b), and an inverse correlation with the F/C ratio. As these modules were expected to contain candidate genes that might be involved in density-dependent behavioural plasticity, we examined them in more detail. They contained 153 and 187 genes for the head tissue and the thorax tissue, respectively (Supplementary Data Files 12,13). Compared to the whole *piceifrons* transcriptome, both modules were enriched for GO terms involved in metabolism, regulatory processes, and cuticle formation (Fig. 4c). Furthermore, several GO terms involved in the cell cycle were enriched in the thorax module (Fig. 4c).

Within a co-expression module, genes are often involved in one or few molecular pathways, regulated by hub genes, that exhibit higher connectivities and have more partners than other genes in the module^71,72^. For both modules we chose to focus on, a large proportion of these hub genes were hexamerin-like proteins (Fig. 4d). After manually assembling all hexamerin-like proteins from the four *Schistocerca* species, we found nine full-length sequences (Supplementary Fig. S8, Supplementary Table S12) that all exhibited differential expression between isolated-reared and crowd-reared individuals for at least one tissue in *piceifrons* (Supplementary Fig. S9). Given their patterns of co-expression with behaviour in particular, these results suggest that hexamerins might play an important role in density-dependent behavioural plasticity.

## Discussion

Locust phase polyphenism is one of nature’s most striking examples of coordinated alternative phenotypes involving a myriad of density-dependent plastic reaction norms^3,19^. Recent studies have shown that some of these individual reaction norms are not only present in locust species, but also in closely-related non-swarming species^26,30,31,33^. This suggests that they likely evolved independently along the phylogeny^26^, and became coordinated in locust species. However, this inference is mostly based on available literature data^26,29^, and not on a detailed empirical quantification of these reaction norms in a comparative framework. Using the Central American locust and three closely-related non-swarming grasshoppers as a comparative model clade, we have demonstrated that all four species, regardless of whether they swarm in nature or not, exhibit clear density-dependent phenotypic plasticity, but the individual reaction norms varied among species (Figs. 1, 2, 3). We found that density-dependent reaction norms in behaviour (Fig. 1), nymphal colouration (Fig. 2a,b), morphometric ratios (Fig. 2c), and gene expression (Fig. 3) each showed different magnitudes of plasticity among the four species, suggesting that these individual reaction norms did not necessarily evolve in a concerted manner. Furthermore, we did not find a single density-responsive gene that is shared by all four species, and only a low number of genes that were density-responsive in the two most polyphenic species, despite the fact that many of the phenotypic responses to density are expressed in a similar manner (Fig. 3b). Instead, we found that the majority of the genes responded to density in a species-specific manner, even though these density-responsive genes were functionally similar across species. These findings collectively point to the insight that the ultimate expression of locust phase polyphenism is a consequence of the independent evolutionary trajectories of different individual reaction norms that may have different yet functionally similar molecular machineries, which become coordinated.

All New World *Schistocerca* species, including the four species in our study, descended from a *gregaria*-like ancestor that presumably colonised the New World from Africa via transatlantic migration about 6 million years ago^26^. This implies that their common ancestor expressed an extreme form of density-dependent phenotypic plasticity, similar to what is observed in *S. gregaria* today. The adaptive significance of locust phase polyphenism has been abundantly documented for *S. gregaria*^19^, and it can thus be inferred that the ancestral phenotypic plasticity in *Schistocerca* was an evolutionary novelty that could have evolved as an adaptation to heterogenous environmental conditions resulting from the aridification of northern Africa^26^. However, it is not clear whether this ancestral plasticity was still adaptive for the ancestral *Schistocerca* that first colonised the New World and its descendants, which probably encountered very different environments from the African desert. To date, there is only one example of non-swarming *Schistocerca* species that shows adaptive plasticity. A North American birdwing grasshopper, *Schistocerca lineata* Scudder, has many host-plant associated ecotypes^56^, one of which has a close association with toxic *Ptelea trifoliata* and shows density-dependent warning colouration^32^. At low density, nymphs are green, but at high density, they show a combination of yellow and black patterns, which advertises their toxicity^32,73^. It has been hypothesised that the ancestral plasticity, or at least the ancestral genetic variation for expressing density-dependent colour plasticity, could have been retained in sedentary *Schistocerca* species^31,73^, which can become a starting point for the evolution of novel traits. Indeed, many contemporary *Schistocerca* species retain hidden density-dependent phenotypic plasticity, which is difficult to observe in nature, but can be induced in the lab^30,31^. Theoretical studies have shown that the cost of maintaining the genetic variation that underlies phenotypic plasticity may be negligible (reviewed in ref^74^). The adaptive significance of density-dependent phenotypic plasticity in the four species we studied is currently unknown although it is highly likely that locust phase polyphenism is adaptive in the Central American locust, as in the desert locust. Given our findings on differential responses of individual reaction norms across the species, we hypothesise that the environmentally induced developmental switch^5^ enabling the expression of density-dependent plastic reaction norms is ancestral and phylogenetically conserved within *Schistocerca* although downstream gene regulatory networks might have evolved differently across species.

Although many phenotypic plasticity studies have focused on a single trait of adaptive importance that is environmentally plastic, the ultimate expression of phenotypic plasticity often involves multiple reaction norms^2,3^. It is not always clear whether these individual reaction norms evolve in a coordinated fashion or not. Our study shows that density-dependent phenotypic plasticity in *Schistocerca* is not a singular response, but a culmination of many individual reaction norms that respond to the same environmental stimulus—changes in local population density. Furthermore, these individual reaction norms seem to follow different evolutionary trajectories, possibly due to selection-driven genetic accommodation^6^ or genetic drift working independently on different traits^73^. For example, *piceifrons* and *americana* showed nearly indistinguishable reaction norms in their behaviour, size and morphometric ratios, but the former exhibited more plastic reaction norms in nymphal colouration and gene expression than the latter (Figs. 1, 2, 3). In *nitens*, most of the reaction norms we quantified were less plastic compared to other species, but the density-dependent response of the nymphal size seemed identical to the one in *cubense* (Figs. 1, 2, 3). Another insight comes from our WGCNA analysis, where different modules are linked to nymphal coloruation than to behaviour and morphology, suggesting that these traits are regulated by different molecular networks. A more in-depth investigation of the behavioral modules is needed to test whether the same genes influence both behavior and morphology.

Understanding the molecular basis of phenotypic plasticity is the next frontier of plasticity research^5^ and our study offers new insights that can only be obtained from a comparative perspective. We were surprised at the finding that the molecular response to density in these four species seemed to be to a large extent species-specific, without a large overlap of density-responsive genes across species. Initially, we expected that at least some genes would be density-responsive across these four phylogenetically close species, given that they showed similar plastic reaction norms in behaviour and morphology. Instead, we found that the molecular response to density was conserved at the functional level rather than at the level of individual genes, in that density-responsive genes had largely similar functions across species (Fig. 3d, Supplementary Data File 11). This trend seems to also hold true when comparing density-dependent gene expression in *piceifrons* to *S. gregaria*, which likely shows the most ancestral form of locust phase polyphenism in the genus, and *L. migratoria*, which represents an independent origin of locust phase polyphenism as it belongs to a different subfamily^16,24^. For instance, all three of these locust species exhibit large density-dependent differences in metabolic, regulatory and cuticular genes^57–59,63,64,67,75,76^, suggesting the molecular response to different rearing densities is functionally similar among them. Because our study focused on individuals that were isolated or crowded during their entire nymphal development, the density-responsive genes reported here are either downstream effector genes or genes involved in the maintenance of the two phenotypes. It is very well possible that the “master switch” genes that are expressed at the onset of phase change, are more phylogenetically conserved. In addition, we focused on one single timepoint, and sampling more timepoints, especially at the early onset of phase change, could have increased the number of shared density-responsive genes between the different species. Nonetheless, our WGCNA analysis offers an insight that at least part of the underlying molecular machinery of their density-dependent phenotypic plasticity is more conserved, as there are several modules of co-expressed genes that are highly correlated with the quantified reaction norms in behaviour and another set of modules correlated with morphology (Fig. 4b). In particular, we have found a strong case for the involvement of hexamerin-like proteins in density-dependent behavioural plasticity. All nine hexamerin-like proteins reported in our study are well-conserved within the family Acrididae (Supplementary Fig. S9), and several hexamerin-like proteins are influenced by the rearing density in *S. gregaria*^58^ and *L. migratoria*^63,66^ (Supplementary Table S12), suggesting that they might exhibit similar functions in these different locust species. Originally described as storage proteins^77^ and juvenile hormone binding proteins^78–80^, hexamerin-like proteins are in fact highly multifunctional^81^. Several of these functions are in fact dependent on juvenile hormone (JH)^82–84^, a multifunctional hormone known to influence several phase-related characteristics in locusts, including nymphal colouration and reproduction. However, there is only scant evidence for a role of JH as a major regulator of locust phase polyphenism^15,23,85–87^ (but see ref.^88^), although JH has been shown to induce physiological traits that are associated with solitarious phase. Similarly, we were unable to find clear evidence for the role of JH (Supplementary Fig. S7) in the regulation of locust phase polyphenism in *piceifrons*, suggesting hexamerin-like proteins might exert at least part of their functions in locusts through other mechanisms. In addition to the hexamerin-like proteins, there are other genes, including pacifastins and takeout proteins, that are differentially expressed in all three locust species (*piceifrons*, *S. gregaria* and *L. migratoria*) for which molecular data are available up to this point^57,58,61,63,66,89–92^. An even more striking example of a highly conserved molecular mechanism underlying at least one aspect of density-dependent phase polyphenism is [His^7^]-corazonin, which causes nymphal black patterning in *L. migratoria, S. gregaria, piceifrons*, and *americana*^93–95^. We have shown that the evolution of density-dependent phenotypic plasticity in *Schistocerca* has been shaped by both shared ancestry and species-specific processes by focusing on a clade that includes both locust and non-swarming grasshoppers. Then, what makes locusts differ from non-swarming grasshoppers, both of which show similar density-dependent phenotypic plasticity? Our study showed that *piceifrons* and *americana* exhibited nearly identical behavioural polyphenism in the laboratory (Fig. 1, Supplementary Fig. S2, Supplementary Tables S2, S3), to a degree that is highly reminiscent of that of the desert locust^48^. However, *piceifrons* is a swarming locust while *americana* has never been reported to swarm in nature and seems only capable of a small degree of collective movement in the field, even under high densities^34,96,97^. Without further experiments, we cannot entirely exclude the possibility that *americana* is capable of swarming, but it seems unlikely that it has not experienced the environmental conditions conducive to swarm formation so far. The molecular response to rearing density was much larger in *piceifrons* than in *americana*, especially in the thorax (Fig. 3a). In addition, the overlap in density-responsive genes between *piceifrons* and *americana* was relatively low (Fig. 3b), and there were several density-responsive genes that exhibited opposite expression patterns in both species (Fig. 3c). Possibly, the differences between a swarming locust and its closely related non-swarming relatives can be explained by locust-specific responses to density at the molecular level.

Our study highlights the importance of a phylogeny-based comparative perspective and the necessity of integrating the quantification of individual reaction norms with their molecular underpinnings in the study of phenotypic plasticity. We believe that the comparative system we have developed here, which includes multiple species with varying degrees of phenotypic plasticity stemming from ancestral plasticity, will become an important model clade for revealing the significance of plasticity in evolution. Particularly, we think that our emphasis on considering multiple reaction norms that respond to the same environmental stimulus across species is a useful perspective that should be incorporated into other study systems of phenotypic plasticity. The findings of our study set up the stage for future studies focusing on the roles of selection and drift driving the evolution of plastic traits and the molecular mechanisms of genetic accommodation and assimilation^5,9^.

## Methods

### Study organisms and rearing regime

The four species included in this study (*piceifrons, americana*, *cubense*, and *nitens*) were maintained as long-term laboratory colonies in the Department of Entomology at Texas A&M University. The *piceifrons* colony was established from an outbreak population in Yucatan, Mexico collected in October 2015, and imported under a USDA permit (USDA APHIS PPQ P526P-15-03851). The *americana* colony was established from a population in Brooksville, Florida, collected in September 2010. The *cubense* colony was established from a population in Islamorada in the Florida Keys collected in January 2011. Finally, the *nitens* colony was established from a population in Terlingua, Texas, collected in May, 2015. All experiments were performed at least 3 generations after the establishment of the lab colonies. The insects, which originated from crowd-reared parents, were reared under two density conditions (crowded vs. isolated) from hatchlings up to their last nymphal instar as previously described^30,31^. In both density conditions, the insects were reared at 12 h of light and 12 h of darkness at 30 °C, and were fed daily Romaine lettuce and wheat bran. Additional details on the rearing regime are presented in Supplementary Methods.

### Behavioural assays

To quantify the behavioural reaction norms in response to rearing density of last instar nymphs, we used an assay system established for the desert locust by ref^48^, as previously described^30,31^ with a few modifications. The test subject was recorded for 12 min with a video camera suspended above the rectangular arena, after which EthoVision XT 12 (Noldus Information Technology Inc.) software was used to video-track the behavior. The arena was divided into three equal zones: a stimulus zone, a non-stimulus zone and a neutral zone. We quantified 50 individuals per density condition per species and extracted a total of ten behavioural variables, equally divided between activity-related and attraction-related variables: ‘distance moved (cm)’, ‘movement (s)’, ‘velocity (cm/s)’, ‘rotation frequency’, ‘wall climbing (s)’ ‘stimulus zone time (s)’, ‘neutral zone time (s)’, ‘non-stimulus zone time (s)’, ‘stimulus wall time (s)’, and ‘final distance to stimulus wall (cm)’. These data were first analysed with a nonparametric multivariate analysis using the R package nparMD^98^, with rearing density and species as independent variables. Secondly, we analysed each behavioural variable separately using generalised linear models (GLMs) and a hurdle model approach. We used species, density, and the interaction between them as independent variables for each GLM. Lastly, we determined the overall similarity in behaviour among all of the density-species treatment pairs using the hypervolume package^99^. Additional details on the behavoural assays are presented in Supplementary Methods.

### Analysis of the black patterns, body size and morphometric ratio

The extent of the black patterns was quantified from 30 last instar nymphs for each species and rearing condition. For each specimen, we captured images of the frontal view of the head, the lateral view of the pronotum, the left hind femur and the left wingpad. The extent of the black pattern in each body part was quantified using the R package patternize^55^, as previously described in ref^31^. For each species and rearing condition, the length of the pronotum, the length of the hind femur (F) and the maximum width of the head (C) were measured from 15 last instar males and 15 last instar females in ImageJ, after which the F/C ratio was calculated. Statistical significance was assessed with a three-way ANOVA, with Tukey’s test as a post-hoc test. Additional details are presented in Supplementary Methods.

### RNA extractions, sequencing and transcriptome assembly

RNA extractions, RNA sequencing, and transcriptome assembly were described before^100,101^. In short, RNA was extracted for five specimens, three days after moulting to their final instar, for each rearing condition and species using a Trizol-chloroform extraction, followed by clean-up with a RNeasy mini kit (Thermofisher Scientific). Library preparation with Illumina’s TruSeq Stranded Total RNA Library Prep Kit, paired-end sequencing (150 bp) using 8.5 lanes on an Illumina HiSeq4000 (San Diego), and pre-processing steps were performed at Texas A&M AgriLife Research Genomics and Bioinformatics Service. After filtering reads with Trimmomatic^102^ and FastQ Screen^103^, they were used for transcriptome assembly using Trinity v2.2.0^104^. Transcriptomes were assembled separately for the head and thorax tissue of each species, resulting in eight different transcriptomes. Transcriptomes were filtered using CD-hit-EST^105,106^ with a threshold of 0.9, and Transrate^107^ using the suggested cut-off. They were annotated using a Blast2GO-plugin^108^ in Geneious environment (R10.2.6; BioMatters, Ltd). Additional details are presented in Supplementary Methods.

### Gene expression analysis

The differential expression analysis was described before^94^ with some modifications. Reads were mapped back to their respective transcriptomes using Bowtie2^109,110^, after which the idxstats tool from Samtools^111^ was used to generate count files. Differential expression analysis was performed using both edgeR^68^ and DEseq2^69^. To assess the overlap of density-responsive genes between the different species, the reads from the head and thorax tissues of each species were mapped to the head and thorax transcriptome of *piceifrons*, respectively, as described above. We subsequently used the weighted gene co-expression network analysis (WGCNA)^71^ to analyse transcript co-expression patterns and calculate the correlation between the eigengene of each module and a set of representative phenotypic traits (distance moved, stimulus zone time, and non-stimulus zone time, female pronotum length, female F/C and the first principal component of the PCA of the black patterns). For this analysis, the phenotypic values of the sequenced individuals were inferred from the averages of each group of measured individuals. Enrichment of gene ontology terms for sets of differentially expressed genes as well as for WGCNA modules was analyzed with fatiGO^112^, which is integrated in Blast2GO. Hexamerin and hemocyanin sequences for *L. migratoria*, *S. gregaria* and other orthopterans were obtained from Genbank (https://www.ncbi.nlm.nih.gov/genbank/), Kang et al.^66^ and Locustmine^63^, and were used to extract hexamerin-like proteins from our eight transcriptomes with Megablast and tblastx in Geneious. These sequences were subsequently manually curated to obtain full length sequences for each species. Additional details are presented in Supplementary Methods.

## Supplementary Information

Supplementary Information.

## Data Availability

All BioSample information and raw sequencing data can be found under BioProject PRJNA633949 on the NCBI website. The transcriptomes produced during this project were deposited as Transcriptome Shotgun Assembly projects at DDBJ/ENA/GenBank under the accessions GIOR00000000, GIOS00000000, GIOT00000000, GIOU00000000, GIOV00000000, GIPC00000000, GIOW00000000, and GIPD00000000. The versions described in this paper are the first versions, respectively GIOR01000000, GIOS01000000, GIOT00000000, GIOU01000000, GIOV01000000, GIPC01000000, GIOW01000000, and GIPD01000000. All sequences for hexamerin-like sequences were deposited in GenBank, and the associated accession numbers can be found in Supplementary Table 13. All downstream datasets generated and/or analysed during this study have been published in Dryad Digital Repository [https://doi.org/10.5061/dryad.dz08kprwz]. Furthermore, additional information related to methods and analyses are included in Supplementary Information.

## Code availability

All R scripts for the statistical analyses and bioinformatics haven been published in Dryad Digital Repository [https://doi.org/10.5061/dryad.dz08kprwz].
