# Supplementary material for: Comparative analysis of phenotypic plasticity sheds light on the evolution and molecular underpinnings of locust phase polyphenism
Source: Sci Rep. 2021 Jun 7;11:11925. doi: 10.1038/s41598-021-91317-w (PMC8184943; doi:10.1038/s41598-021-91317-w)
Supplement: Supplementary file 1 — Supplementary Information. [file 41598_2021_91317_MOESM1_ESM.pdf]

Supplementary Information

Comparative analysis of phenotypic plasticity sheds light on the evolution of locust phase polyphenism

Bert Foquet, Adrian A. Castellanos, and Hojun Song

Table of Contents

SUPPLEMENTARY METHODS **2**

Study organisms and rearing regime *2*

Behavioural assays *2*

Statistical analysis of behavioural reaction norms *4*

Analysis of black patterning *4*

Analysis of body size and morphometric ratio *5*

RNA extractions *5*

Sequencing and read processing *5*

Transcriptome assembly and annotation *6*

Differential expression analysis *6*

Co-expression network analysis *7*

Hexamerin sequence analysis *7*

Data Availability statement *8*

SUPPLEMENTARY FIGURES **9**

SUPPLEMENTARY TABLES **18**

SUPPLEMENTARY DATA FILES AND DESCRIPTIONS PROVIDED VIA THE DIGITAL REPOSITORY DRYAD **31**

SUPPLEMENTARY REFERENCES **37**

SUPPLEMENTARY METHODS

Study organisms and rearing regime

We used four closely related species in the genus *Schistocerca* Stål, maintained as long-term laboratory colonies in the Department of Entomology at Texas A&M University. The four species were: Central American locust, *S. piceifrons* (Walker) and three non-swarming grasshoppers, *S. americana* (Drury), *S. serialis cubense* (Saussure), and *S. nitens* (Thunberg). The *piceifrons* colony was established from an outbreak population in Yucatan, Mexico collected in October 2015, and imported under a USDA permit (USDA APHIS PPQ P526P-15-03851). The *americana* colony was established from a population in Brooksville, Florida, collected in September 2010. The *cubense* colony was established from a population in Islamorada in the Florida Keys collected in January 2011. Finally, the *nitens* colony was established from a population in Terlingua, Texas, collected in May, 2015. All experiments were performed at least 3 generations after the establishment of the lab colonies. The insects were reared under two density conditions, similar to the rearing regime previously described[1,2]. To induce the solitarious phase, we isolated hatchlings into individual plastic cages (10.16 x 10.16 x 25.4 cm) and reared them singly until the last instar nymphal stage. The individual cage was connected to a positive filtered airflow from the top, and designed in a way that the insect reared inside was physically, visually, and chemically isolated from each other. To induce gregarious phase, we reared the insects in high density from hatchings to the last instar nymphs. Both cage size and the number of individuals used for crowding depended on the species. For *piceifrons*, about 250 individuals were kept in a large cage (40.64 x 34.29 x 52.07 cm). For *americana* and *cubense*, 150-200 individuals were kept in larger cages (30.48 x 35.56 x 50.8 cm). For *nitens*, over 50 individuals were kept together in a small cage (30.48 x 35.56 x 50.8 cm) because we observed that the insects would die in the density used for other species. For RNA sequencing experiment, the isolation treatment was identical to what was described above, but for the crowding treatment, we placed 25 penultimate crowd-reared nymphs in small mesh cages (13.21 x 13.21 x 22.86 cm) and reared them until they molted to the last instar, which were then used for the experiment. In both density conditions, the insects were reared at 12 hours of light and 12 hours of darkness at 30°C, and were fed daily Romaine lettuce and wheat bran.

Behavioural assays

To quantify reaction norms in behaviour in response to rearing density, we used the assay system established by Roessingh, et al. [3] initially for the desert locust. In short, the assay utilized a rectangular arena (57 × 31 × 11 cm) with a chamber at each end: an empty non-stimulus chamber to simulate a low-density condition and a stimulus chamber containing 30-50 conspecific last instar nymphs to simulate a high-density condition. For each assay, a test subject, reared in either isolated or crowded condition, was placed into a blackened 50 ml Falcon tube for two minutes to reduce the effect of handling and then slowly introduced into the arena via a hole in the center of the arena floor. A video camera suspended directly above the arena was used to record behavioural responses of the test subject to the stimuli for 12 minutes. We performed this assay 3–5 days after nymphs molted to the last nymphal instar, as previously described [1,2] with a few modifications. We quantified 50 individuals per density condition per species, thus a total of 400 individuals. For *piceifrons*, we used either a Panasonic HDC-TM900 (704x480, 29.97 frames/second) or a Basler acA1300 – 60gc (1280x1024, 60 frames/second), while for *americana, cubense,* and *nitens*, the assays were recorded with either a Basler acA1300

(720x480, 29.97 frames/second) or a Basler acA1300 – 60gc (1280x1024, 60 frames/second). We used EthoVision XT 12 (Noldus Information Technology Inc., Leesburg, VA) software to video-track behaviour, analysing 12 minutes of each assay at a frame rate of 30 frames per second. The arena was divided into three equal zones using the EthoVision software: a stimulus zone (the third adjacent to the stimulus chamber), a non-stimulus zone (the third adjacent to the non-stimulus chamber), and a neutral zone (the central third). We also designated all four walls (top, bottom, left, right) as a wall zone to measure climbing activity. The optimized detection settings differed for each species and camera used, but consistently used 'differencing' as a detection method. The subject colour was set as 'darker and lighter' than the background, so that both light-coloured and dark-coloured individuals could be detected, except for *nitens*, where the optimal detection was obtained when choosing 'darker'. Sensitivity and contour erosion/dilation values were highly dependent on both the species and the camera the videos were taken with. For each behavioural assay, raw position data were acquired and all tracks were manually inspected and corrected where necessary. Before exporting data, tracks were smoothed using the 'Minimum Distance Moved' (MDM) filter. This filter removes inherent noises from the video tracks, so that only true movement is captured. It was optimized based on the effect of different MDMs on the 'distance moved' variable, as noted in a previous study[4]. For all species in this study, the optimal MDM was 0.2 cm. Filtering by the MDMs resulted in filtering out actual movement in certain frames, leading to a spotty pattern of movement, and as a direct result, several dependent variables that EthoVision output as default, including 'heading', 'turn angle', 'meander', 'mobility', and 'angular velocity', were deemed to not accurately represent the behavioural variables for the test subjects, and were thus excluded from subsequent analyses. After excluding these variables, we kept a set of five variables as activity-related variables in our final analysis: 'distance moved' describing the distance moved in centimeters, 'movement' describing the time spent moving in seconds, 'velocity' describing the average velocity (cm/second) of the test subject, 'rotation frequency' describing the number of rotations and 'wall climbing' describing the time spent (in seconds) in any of the arena zones designated as wall. For two of these variables, we made the following adjustments before statistical analyses. The 'movement' was calculated as the number of frames representing movement above a certain velocity threshold. To cope with the disjointed pattern of movement caused by the MDM-filter, the 'movement' variable was further optimized by visually comparing it to the actual video. The best estimate of actual movement was found by averaging the movement over 10 samples. The 'velocity' variable exported by EthoVision did not accurately assess the actual average velocity during the assay (see Kilpatrick, et al. [2]), so we calculated a new 'velocity' variable by manually dividing the 'distance moved' by the 'movement'. At last, a total of five attraction-related behavioural variables were exported: 'stimulus zone time' describing the time spent (in seconds) in the third of the arena close to the stimulus and the walls around it, 'neutral zone time' describing time spent (in seconds) in the middle third of the arena and its walls, 'non-stimulus zone time' describing time spent (in seconds) in the last third of the arena closed to the non-stimulus chamber and the walls around it, 'stimulus wall time' describing the time spent (in seconds) on the wall facing the stimulus chamber, and 'final distance to stimulus wall' describing the linear distance (in cm) between the test subject and the stimulus wall, bringing the total of behavioural variables to ten.

Statistical analysis of behavioural reaction norms

To test whether the behavioural responses differed among the species and the density treatment combinations, we used the following statistical methods. First, we used nonparametric multivariate analyses as implemented in the R package nparMD[5]. The behavioural data we collected were often resistant to transformation and highly skewed by zero values, so these nonparametric techniques enabled us to analyse these non-normal data. We split analysis of our behavioural variables according to measurement unit (i.e., separate analyses for seconds and centimeters). Secondly, we analysed each behavioural variable separately using generalized linear models (GLMs). Due to the aforementioned zero inflation found in several of these behavioural variables, we used a hurdle model approach that first used logistic regression of zero and nonzero data and then a model for all of the nonzero data. We used species, density, and the interaction between species and density as independent variables for each GLM. During the logistic regression step of the hurdle model, we partitioned data into nonzero and zero categories and used a binomial distribution. As our nonzero data were continuous, positive, and skewed, we used a gamma distribution with a log link for the second part of our hurdle models. All results were adjusted for multiple comparisons using the Bonferroni correction. Lastly, we determined the overall similarity in behaviours among all of the density-species treatment pairs. We reduced the number of behavioural parameters with a principal components analysis (PCA) and used the first three axes totaling 69.59% of the variation to create hypervolumes for each species in the overall, isolated, and crowded situations using the hypervolume package[6]. For each hypervolume, we calculated pairwise distance to centroid and similarity according to the Sorenson index. We created three sets of hypervolumes for these comparisons: one using all behavioural values, one using only behavioural values from the crowded individuals, and one using behavioural values from the isolated individuals. All statistical analyses were performed in R version 3.3.3[7] with all results included in an RMarkdown output file (Supplementary Data Files 15-16).

Analysis of black patterning

The amount of black patterning was quantified from 30 individuals for each species and rearing condition. Isolated-reared and crowd-reared nymphs were frozen in their last nymphal instar and stored at -20°C. High-resolution digital images were taken with a LK Imaging System (Visionary Digital) with SpyderCHECKR 24 software for colour correction (Datacolour, Lawrenceville, NJ). For each specimen, images of four different body parts were captured: the frontal view of the head, the lateral view of the pronotum, the left hind femur and the left wing pad. The extent of black patterning in each body part was quantified using the R package patternize[8] in R version 3.6.2 (R Core Team 2017), as previously described in Kilpatrick et al.[2]. Patternize is a pattern identification program that can define homology between patterns across specimens either through manually placed homologous landmarks or through the simple alignment of the patterns themselves[8]. Subsequently, it uses various image manipulation techniques to obtain the exact distribution of each colour of interest[8]. Each digital image was first reduced in size to a JPEG file with a width of 1,000 pixels, after which the polygon tool in ImageJ[9] was used to place between eight and 12 landmarks on each of the investigated body regions, which were saved as xy-coordinates. Additionally, the outlines of the different structures were saved as xy-coordinates by drawing their complete circumference in one representative image in ImageJ, again using the polygon tool. Using the set of landmarks and the respective structure outline, images were aligned with the PatLanRGB-function of patternize, and heatmaps

of the extent of black patterning were generated separately for each body region, species and rearing condition by using a set RGB-value of (0,0,0) with a cutoff of 0.15. Finally, a linear PCA was used to better show clustering of specimens. First, patternize generates specimen-specific matrices of pixel coordinates in which coordinates that have the colour of interest have a value of one, while all other coordinates are assigned a value of zero. Subsequently, the variance–covariance matrix obtained from these binary matrices are used for a PCA that also allows visualizing the main variations in colour patterns between samples, as well as showing the predicted colour pattern changes along the principal component (PC) axis[8].

Analysis of body size and morphometric ratio

For each species and rearing condition, three linear dimensions were measured from 15 males and 15 females. Images were calibrated in ImageJ using a ruler present in each image and three independent measurements were performed for each linear dimension, after which the average was used in all further analyses. The three measured dimensions were the length of the pronotum, the length of the hind femur (F) and the maximum width of the head (C). Additionally, the F/C ratio was calculated because this morphometric ratio is considered a reliable predictor of phase in locusts[10,11]. For the linear dimensions, statistical significance was assessed with a three-way ANOVA, with Tukey's test as a post-hoc test in R version 3.6.2 (R Core Team 2017).

RNA extractions

Isolated-reared and crowd-reared female nymphs were marked on their abdomen with a ceramic marker after molting to their last nymphal instar, and were dissected around 72 hours later. The ceramic marker did not have any influence on the behaviour (unpublished observation) and the marked tissues were not used for RNA extractions to further eliminate any unforeseen effect on the experiment. Only the specimens that molted in the morning were used, and all dissections occurred between 8 and 9 AM. Head and thorax tissues were preserved in RNAlater (Qiagen, Valencia, CA) at -20°C, following the manufacturer's guideline. A total of 40 specimens (5 biological replicates for each density condition x 4 species) were dissected for each tissue, bringing the total number of sequenced samples to 80. RNA was extracted using a Trizol-chloroform extraction, followed by clean-up with a RNeasy mini kit using an on-column DNAse treatment with an RNAse-free DNAse set (Thermofisher Scientific, Waltham, MA, and Qiagen, Valencia, CA). RNA concentrations were measured with a spectrophotometer (DS-11, DeNovix, Wilmington, DE), and RNA integrity was analysed with a Fragment Analyser (Agilent Technologies, Ankeny, IA). RNA extracts were only used for sequencing if the ratios of absorbance values of 260 to 280 nm and of 260 to 230 nm were above 2[12], and if RNA Quality Number (RQN) values were over 3.9[13-16].

Sequencing and read processing

Library preparation, sequencing, and pre-processing steps up to demultiplexing base call files were all performed at Texas A&M AgriLife Research Genomics and Bioinformatics Service. For library preparation, Illumina's TruSeq Stranded Total RNA Library Prep Kit was used and paired-end sequencing (150 bp) was performed using 8.5 lanes on an Illumina HiSeq4000 (San Diego, CA). Cluster identification, quality prefiltering, base calling and uncertainty assessment happened in real time with Illumina's software (HCS 2.2.68; RTA 1.18.66.3; default parameter settings). Demultiplexing base call files and formatting them into

FASTQ files was carried out using Illumina's bcl2fastq script (version 2.17.1.14). For further processing, raw reads were imported into a personalized Galaxy environment[17] on a supercomputing cluster of the High-Performance Research Computing group of Texas A&M University (Ada, https://hprc.tamu.edu). We transformed reads to Sanger format with FastQ Groomer[18] and filtered them using Trimmomatic[19]. In Trimmomatic, bases were trimmed at both ends if their quality score was lower than 30, whole reads were trimmed with a sliding window of 3 bases and a minimum average quality score of 30, and finally all reads of less than 30 bp were discarded. Subsequently, FastQ Screen[20] was used to filter out reads from bacterial and other contaminating sources (UniVec core (June 6, 2015), PhiX (NC_001422.1), Illumina adapters, *Gregarina niphandrodes* genome (GNI3), *Encephalitozoon romaleae* genome (ASM28003v2), *Escherichia coli* genome (K12), *Methylobacterium* sp., *Bosea* sp., *Bradyrhizobium* sp., *Klebsiella pneumoniae*, *Sphingomonas* sp., *Rhodopseudomonas* sp. and *Propionibacterium acnes*). We also used FastQ Screen to assess the percentage of reads mapping to genomes of related species; consistently about 40% of all reads mapped to the genome of the migratory locust *Locusta migratoria*, while 5% mapped to the termite *Zootermopsis nevadensis*. Less than 5 % of all reads mapped to the human genome. Reads were re-paired after FastQ Screen using a Galaxy-tool based on the repair.sh script of bbtools (https://sourceforge.net/projects/bbmap/files/). The amount of reads for each sample, before and after filtering, can be found in Supplementary Table 11.

Transcriptome assembly and annotation

The filtered reads were used for transcriptome assembly using Trinity v2.2.0[21] (default settings, *in silico* normalization using the default value of 50 as max read coverage enabled). Transcriptomes were assembled separately for the head and thorax tissue of each species, resulting in eight different transcriptomes. Similar contigs were removed using CD-hit-EST[22,23] with a threshold of 0.9. An additional filtering step was performed with Transrate [24], filtering out all contigs scoring under the suggested cut-off. The quality of these final transcriptomes was analysed with Trinitystats[21] and BUSCO (Benchmarking Universal Single-Copy Orthologs)[25] by comparing to single-copy orthologs of the lineage Insecta. The fraction of reads mapping back to their transcriptome was obtained with bowtie2[26,27] in the preset mode 'very sensitive, end-to-end' and flagstat[28-30]. We subsequently imported all eight final transcriptomes into a Geneious environment (R10.2.6; BioMatters, Ltd, Auckland, New Zealand), where they were annotated using a Blast2GO-plugin[31]. CloudBlast from Blast2GO was used to blast transcriptome sequences to the nr-database for arthropods. Mapping to protein domains was performed with Interproscan[32,33] on the supercomputing cluster of the High-Performance Research Computing group of Texas A&M University (Ada, https://hprc.tamu.edu). The RepeatMasker Web server[34] with RMBlast and 'other arthropods' selected, was used to discover repeats and transposons in our transcriptomes.

Differential expression analysis

Reads were mapped back to their respective transcriptomes using Bowtie2 in the preset mode 'very sensitive end-to-end alignment', with 'no-mixed behaviour' and 'no-discordant behaviour' disabled. We filtered the resultant mapping files with a threshold quality score of 3. The idxstats tool from Samtools[29] was used to generate count files. Differential expression analysis was performed with SARTools[35], using both edgeR[36] and DEseq2[37], in R 3.5.3[7]. For all three programs, all settings were kept at their defaults; the expression in crowd-reared

individuals was compared to that of conspecific isolated-reared individuals. As such, we did a total of eight different comparisons. Five samples were removed from all further analysis after a preliminary investigation of gene expression. One crowd-reared and one isolated-reared *cubense* thorax samples were removed as they showed almost identical gene expression, suggesting possible contamination between the two. Furthermore, two crowded *nitens* thorax samples and one crowded *nitens* head sample were identified as extreme outliers, and thus removed. Thus, a total of 39 head samples and 36 thorax samples were used for all further analyses. Genes with an adjusted p value of ≤ 0.05 and an absolute value $\log_2$foldchange ≥ 1 in either one of the two programs were considered to be differentially expressed.

In an attempt to assess the overlap of differentially expressed genes between the different species, the reads from the head and thorax tissues of all species were mapped to the head and thorax transcriptome of *piceifrons*, respectively, using bowtie2 with settings as described above. Production of count files and discovery of differentially expressed genes were performed as described above. The overlap between differentially expressed genes in different species was assessed using an adapted Fisher exact test in the SuperExactTest package[38] in R.

Co-expression network analysis

We used weighted gene co-expression network (WGCNA) analysis[39] to analyse transcript co-expression patterns in the 75 samples. This method allows the clustering of genes with similar expression patterns in the analysed samples into modules, that are expected to share pathway membership or other functionalities[39]. Read counts for each sample, obtained by mapping reads to the appropriate *piceifrons* transcriptome, were normalized using the TPM (transcripts per million) method in R. Contigs for which the average raw read count was under 5 were removed, after which we retained only the top 60% of contigs with the most variable expression. Remaining contigs were used to generate a one-step unsigned co-expression network. Based on the scale-free topology criterion[40], we set the soft-threshold power for calculating the adjacency matrix to 14 and 8 for respectively head and thorax, both resulting in an R^2 value of over 0.8. Subsequently, genes were hierarchically clustered based on the TOM (Topological Overlap Measure)-based dissimilarity. Modules were detected using DynamicTreeCut[41], with a minimum module size of 30 and values of respectively 0.2 and 0.15 for head and thorax to separate branches in the dendrogram. Subsequently, we calculated the correlation between the eigengene of each module and several behavioural variables. The values for the behavioural variables (‘distance moved’, ‘stimulus zone time’, and ‘non-stimulus zone time’) were the average values obtained for these variables from the 50 specimens of its respective species and density treatment. In addition, we created an arbitrary variable named ‘swarming’, for which we gave a value of ‘1’ for all crowd-reared nymphs of *piceifrons*, and a value of ‘0’ for all others, as only *piceifrons* is the swarming locust. Gene networks were visualized in VisANT visualization software[42], which was also used to select hub genes. Enrichment of gene ontology terms for sets of differentially expressed genes as well as for WGCNA modules was analysed with fatiGO[43], which is integrated in Blast2GO.

Hexamerin sequence analysis

Hexamerin and hemocyanin sequences for *L. migratoria*, *S. gregaria* and other orthopterans were obtained from Genbank (https://www.ncbi.nlm.nih.gov/genbank/), Kang, et al. [44] and Locustmine[45] (http://www.locustmine.org: 8080/locustmine), and were imported in Geneious. Using a combination of Megablast and tblastx with default settings in Geneious, we

obtained hexamerin-like sequences from our eight transcriptomes. The obtained sequences were aligned using MUSCLE version 3.8.24[46], again with default settings, followed by manual curation to obtain full length sequences for each species. To examine the patterns of evolution for the nine hexamerin-like proteins that we obtained from the four *Schistocerca* species, we first aligned these 36 sequences with the 58 orthopteran hexamerin and hemocyanin sequences based on the conservation of amino acids using MUSCLE in Geneious. The aligned matrix (94 terminals and 2,933 aligned nucleotides) was analysed in a maximum likelihood framework by applying the GTRCAT model using RAxML 8.2.12[47] on XSEDE (Extreme Science and Engineering Discovery Environment, https://www.xsede.org) through the CIPRES Science Gateway[48]. Nodal support was evaluated using 1,000 replications of rapid bootstrapping implemented in RAxML. A summary of these sequences, their orthologues in *S. gregaria* and *L. migratoria*, and the Genbank accession numbers for the four species included in this study can be found in Supplementary Table 13.

Data Availability statement

All BioSample information and Raw sequencing data can be found under BioProject PRJNA633949 on the NCBI website. The transcriptomes produced during this project have been deposited as Transcriptome Shotgun Assembly projects at DDBJ/ENA/GenBank under the accessions GIOR00000000, GIOS00000000, GIOT00000000, GIOU00000000, GIOV00000000, GIPC00000000, GIOW00000000, and GIPD00000000. The versions described in this paper are the first versions, respectively GIOR01000000, GIOS01000000, GIOT00000000, GIOU01000000, GIOV01000000, GIPC01000000, GIOW01000000, and GIPD01000000. All sequences for hexamerin-like sequences were deposited in GenBank, and the associated accession numbers can be found in Supplementary Table 13. All raw data used for the analyses of behaviour, nymphal colouration, morphology, differential gene expression, and correlated gene networks, as well as R scripts used for these analyses have been published in Dryad Digital Repository [https://doi.org/10.5061/dryad.dz08kprwz].

SUPPLEMENTARY FIGURES

Supplementary Figure 1: Graphs showing all behavioural data for crowd-reared and isolated-reared nymphs of all four species.
A series of raincloud plots for attraction-related behaviours (top row) and movement-related behaviours (bottom row) in crowded-reared and isolated-reared nymphs of *piceifrons*, *americana, cubense* and *nitens*. Density curves show the general patterns for each rearing condition, while jittered points detail the observed data.

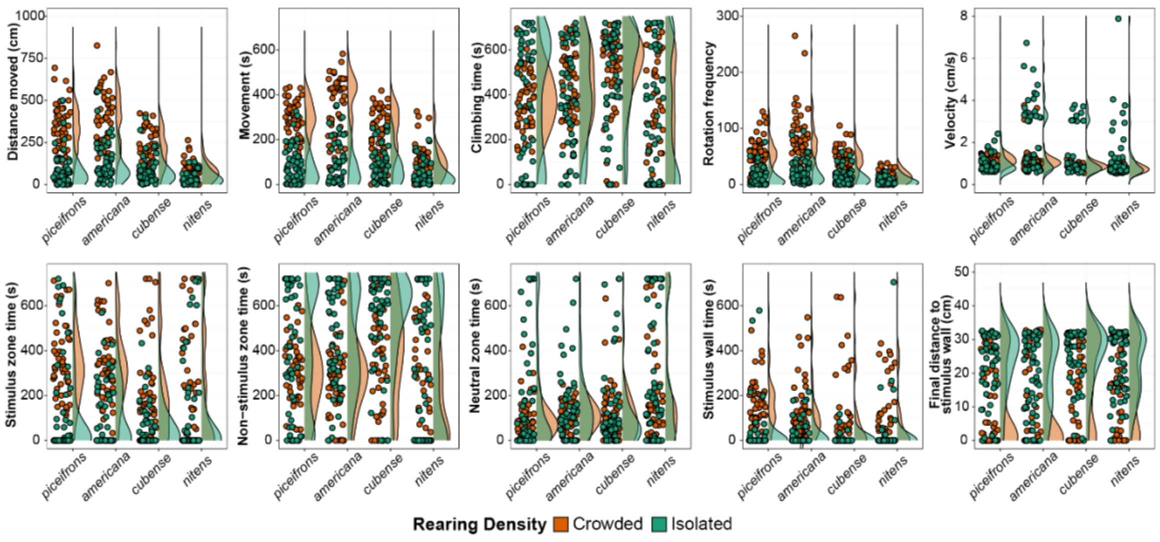

Supplementary Figure 2: Results of the hurdle model for all behavioural variables.
a. Plots showing the results from the logistic regression portions of the hurdle models for all behavioural variables. b. Plots showing the results from gamma distribution portions of the hurdle models for all variables. For both plots, the x-axis depicts the species and species-density interactions, with the isolated-reared condition of *nitens* used as reference. The y-axis depicts the back-transformed model coefficients for each of the model parameters. For a, the y-axis represents the probability of finding a nonzero value for each variable, ranging from 0 to 1. For b, the y-axis represents the expected value of each variable. Error bars depict 95% confidence intervals.

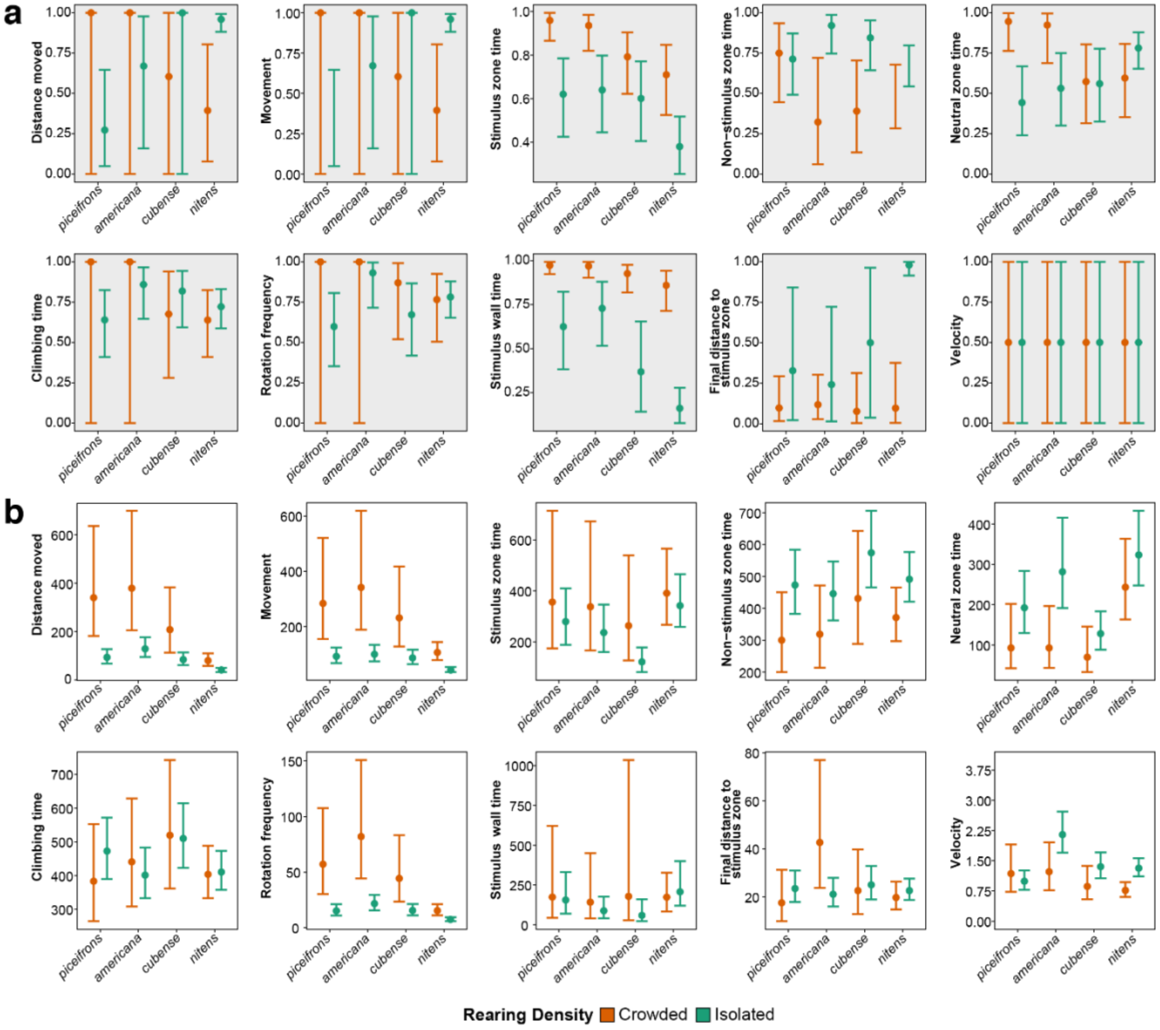

Supplementary Figure 3: Low overlap in density-responsive genes of the head tissue between four different species.

Heatmap showing all genes that show differential expression in the head tissue of at least one of the four species. For all species, the isolated-reared condition was used as reference, and for each gene, the highest log_2foldchange of edgeR or DEseq2 was used. Blue colours represent genes that are downregulated in crowded-reared individuals while yellow colours represent genes upregulated under crowded-reared conditions. Green represents genes for which no significant expression differences were found.

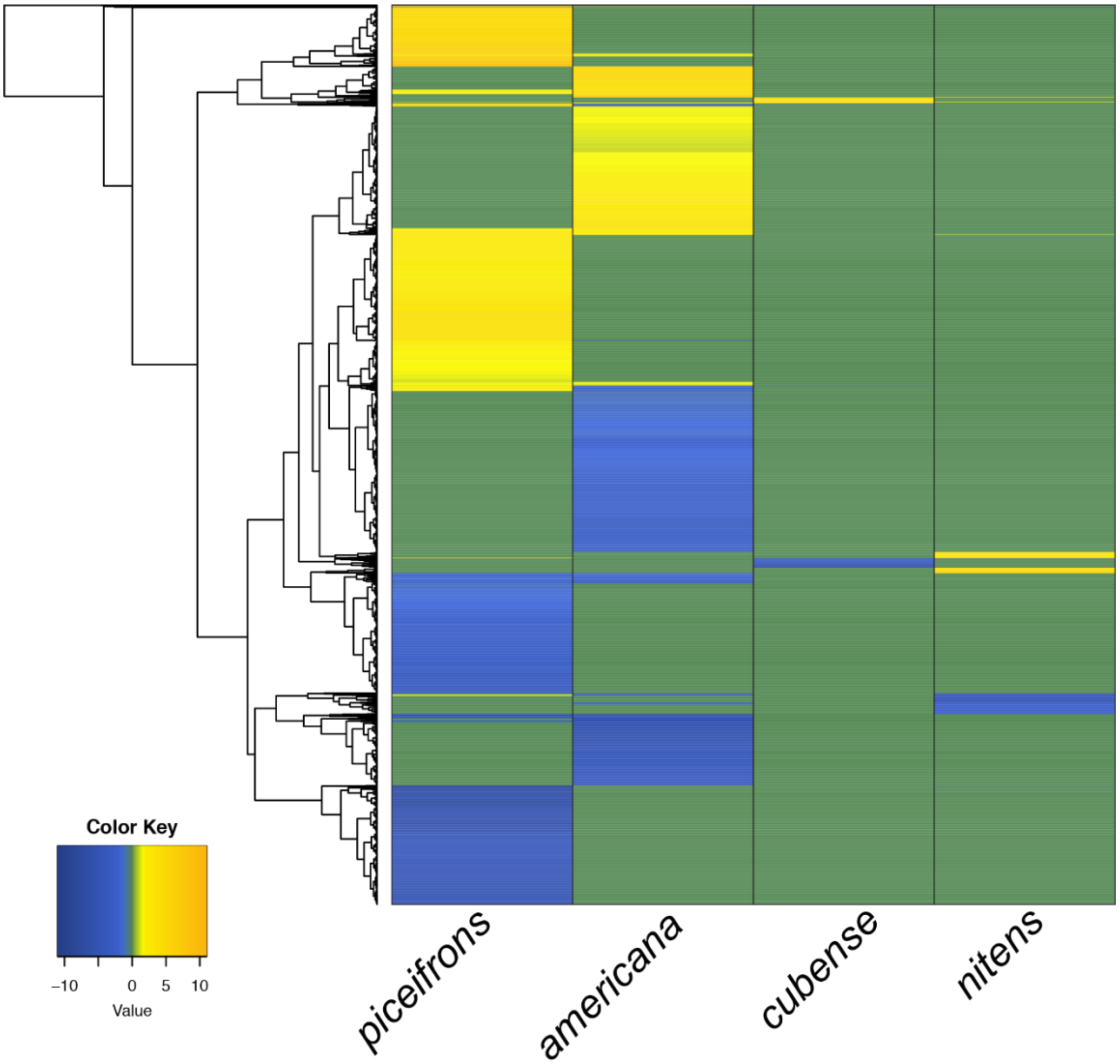

Supplementary Figure 4: Low overlap in density-responsive genes of the thorax tissue between four different species.
Heatmap showing all genes that show differential expression in the thorax tissue of at least one of the four species. For all species, the isolated-reared condition was used as reference, and for each gene, the highest log_2foldchange of edgeR or DEseq2 was used. Blue colours represent genes that are downregulated in crowded-reared individuals while yellow colours represent genes upregulated under crowded-reared conditions. Green represents genes for which no significant expression differences were found.

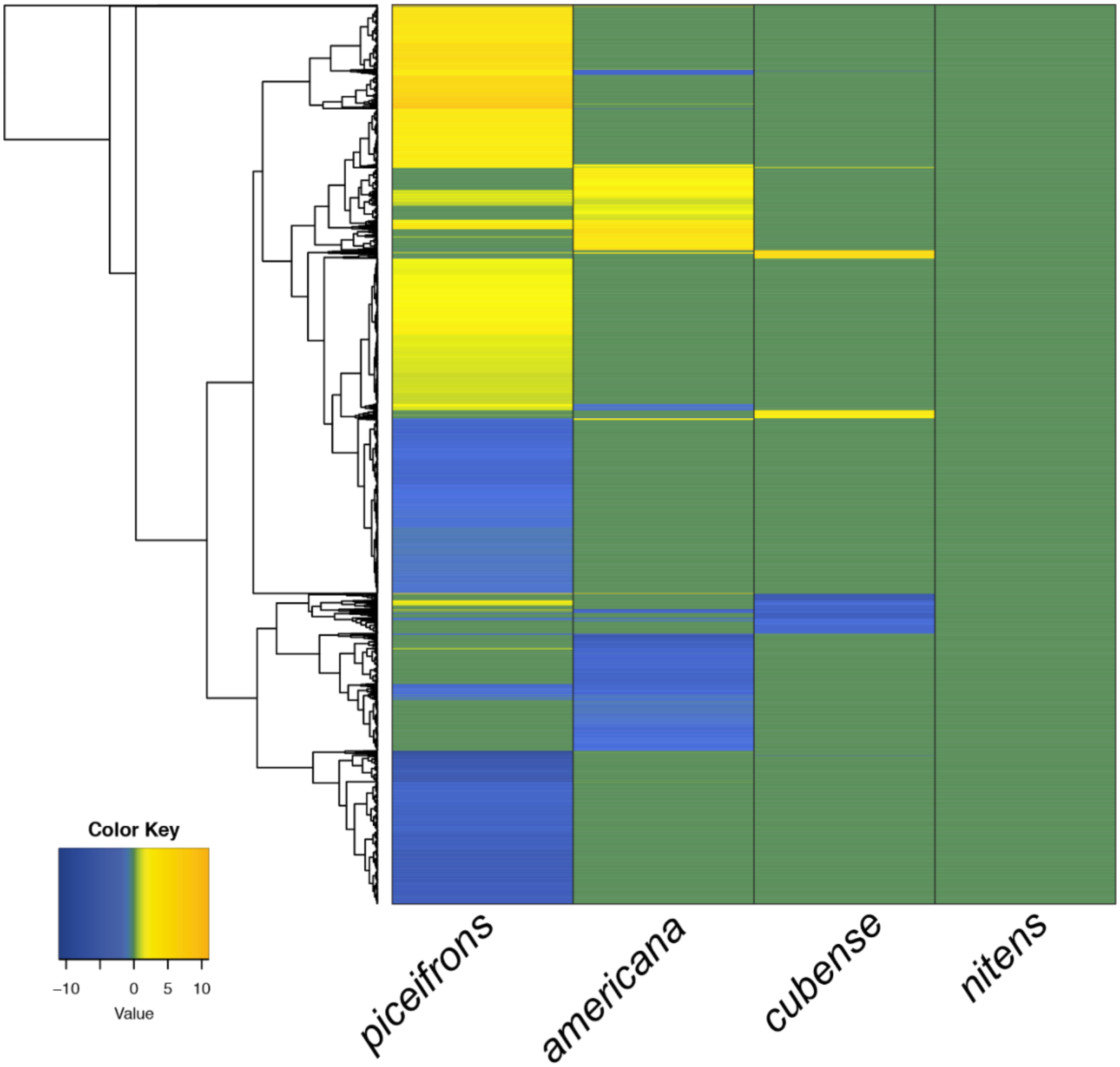

Supplementary Figure 5: WGCNA correlation plot for the head tissue.
Heatplot depicting the correlations between different modules of co-expressed genes of the head tissue and 11 behavioural traits. Stronger correlations are shown by more intense colours, with red signifying a positive correlation and blue an inverse correlation. The upper number in each square represents the correlation value between the eigenvalue of the module and the behavioural trait, calculated by WGCNA, while the lower value refers to the associated *p* value.

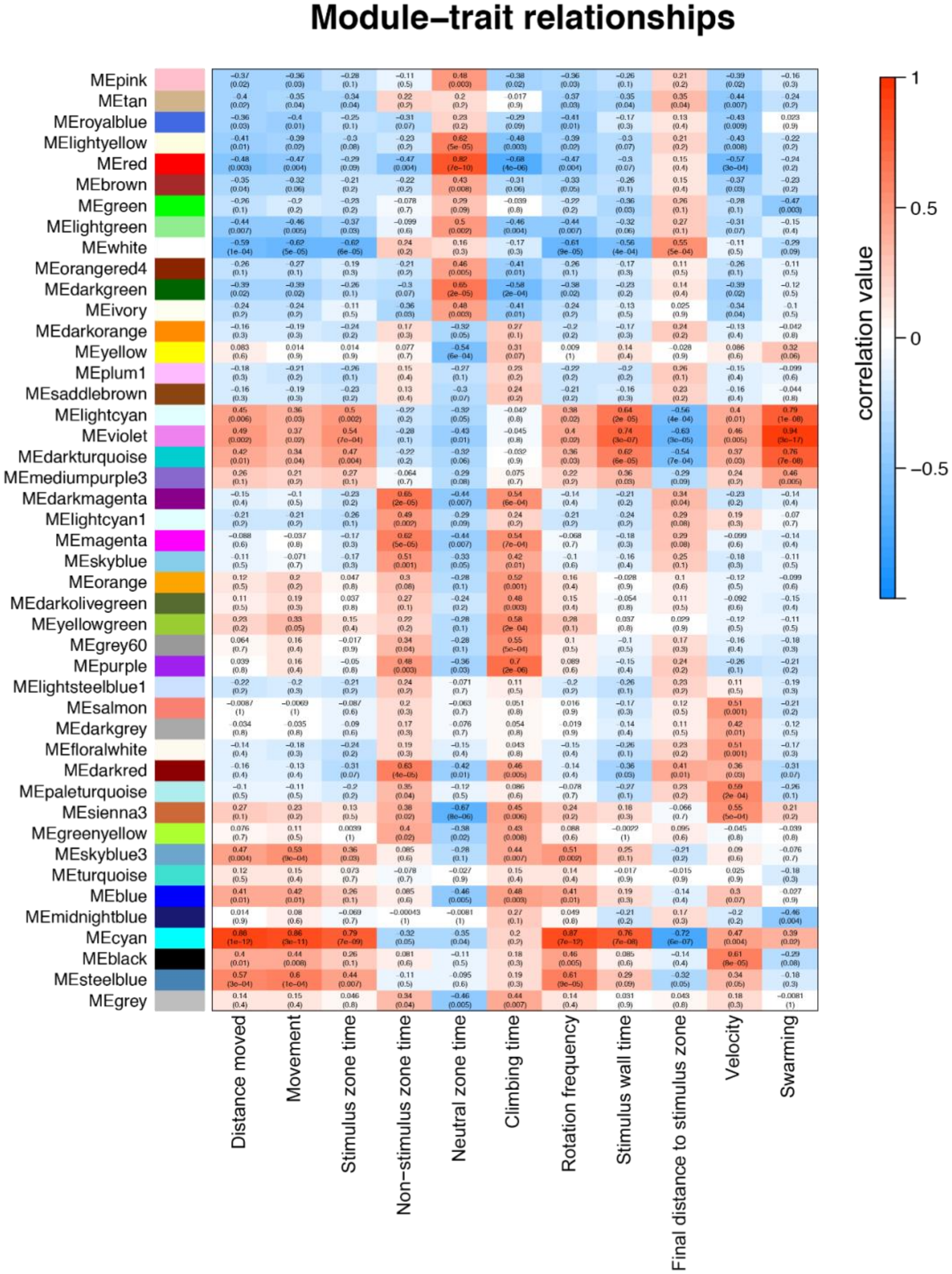

Supplementary Figure 6: WGCNA correlation plot for the thorax tissue.
Heatplot depicting the correlations between different modules of co-expressed genes of the thorax tissue and 11 behavioural traits. Stronger correlations are shown by more intense colours, with red signifying a positive correlation and blue an inverse correlation. The upper number in each square represents the correlation value between the eigenvalue of the module and the behavioural trait, calculated by WGCNA, while the lower value refers to the associated *p* value.

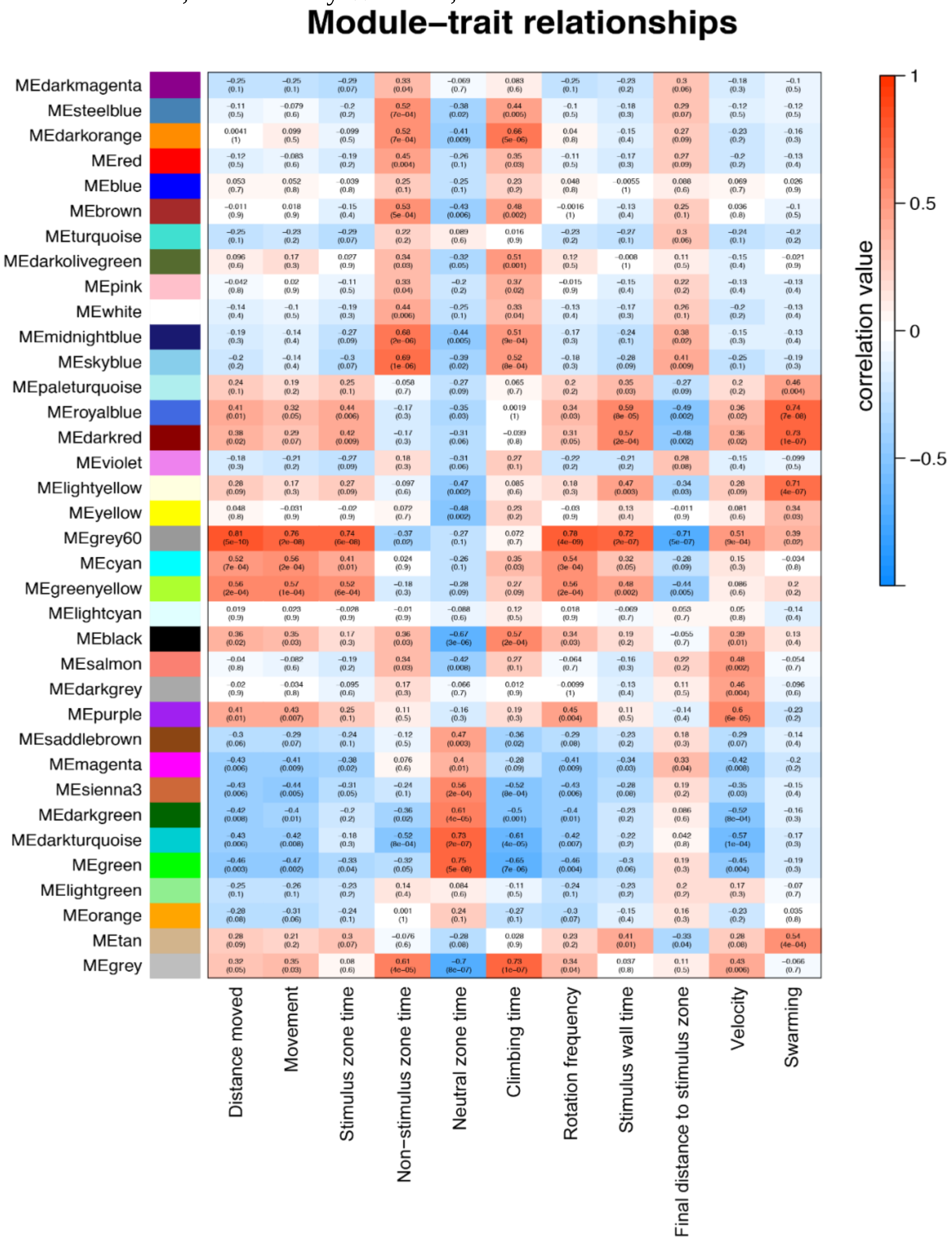

Supplementary Figure 7: Hexamerin gene tree inferred in a maximum likelihood framework. We included all hexamerin-like proteins from our transcriptome data, as well as all known hexamerin sequences of Orthoptera and other representative insects as ingroup. We have recovered all orthopteran species forming a monophyletic group, implying that all orthopteran hexamerin can be traced to a single origin. Within Orthoptera, hexamerin went through many gene expansions, and within *Schistocerca*, we recovered 9 hexamerin-like proteins, shown in magenta, suggesting at least 9 expansion events.

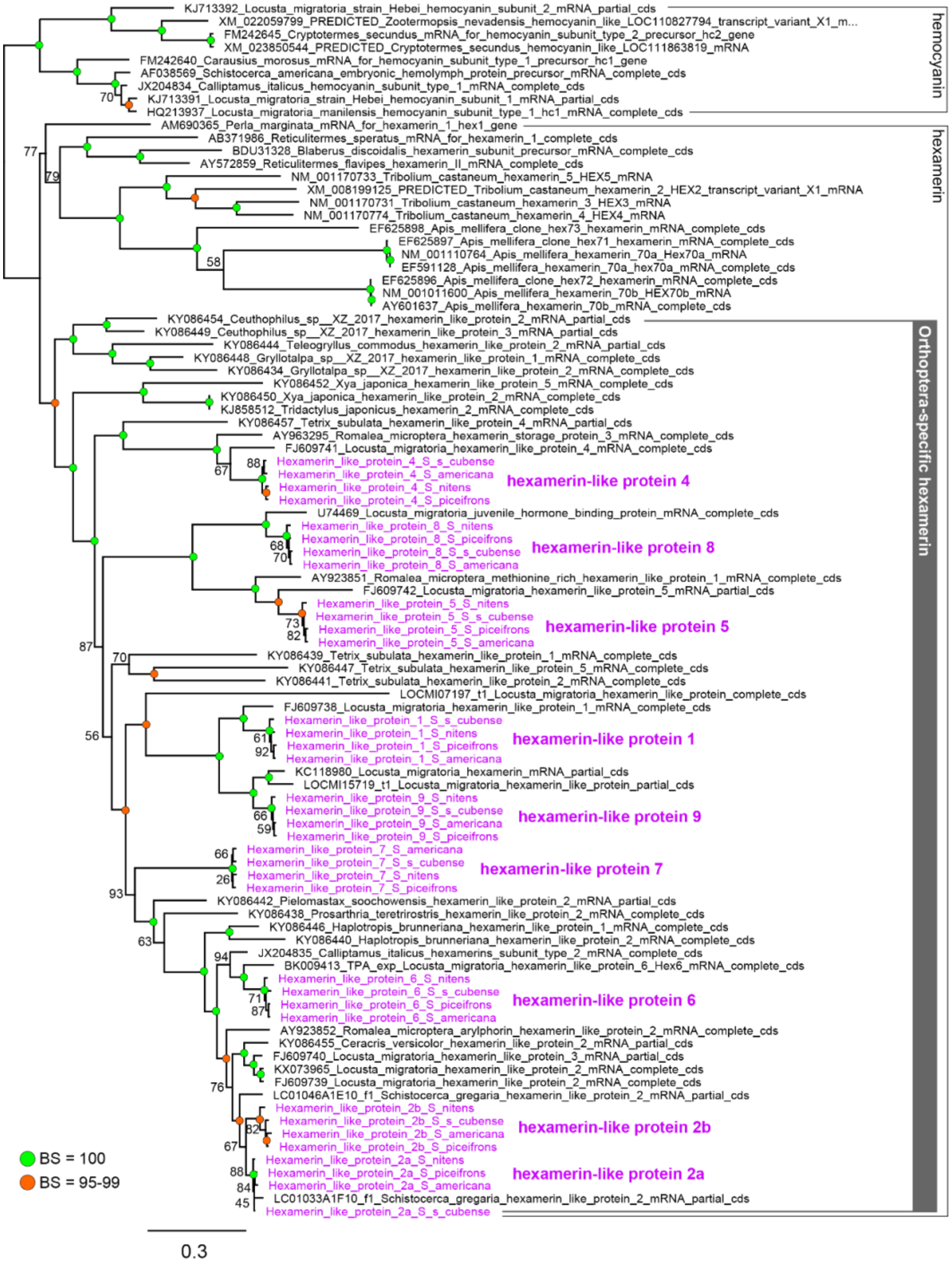

Supplementary Figure 8: Differential expression of hexamerin-like proteins
Graph representing the log_2foldchange values of the 9 hexamerin sequences for both the head tissue and the thorax tissue for all four species. All values are based on the output of DEseq2, with as reference the isolated-reared condition. Significance was based on the adjusted *p* value of DEseq2 (*: $p < 0.05$, **: $p < 0.01$ and ***: $p < 0.001$.)

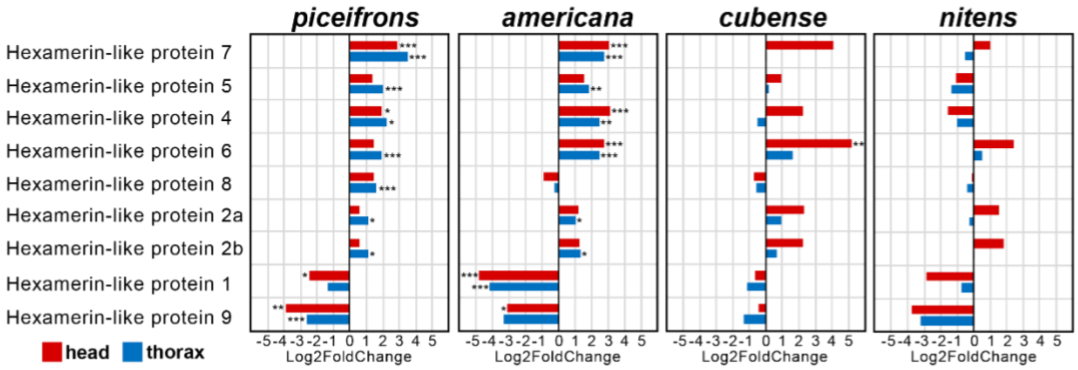

Supplementary Figure 9: Differential expression of JH-related proteins.
Graph representing the $\log_2$foldchange values of genes involved in JH metabolism or known to interfere with or be regulated by JH, for both head and thorax tissue of *piceifrons*. All genes were identified based on reciprocal BLAST hits with annotated nucleotides present in Genbank. All values are based on the output of DEseq2, with as reference the isolated-reared condition. Significance was based on the adjusted *p* value of DEseq2 (*: $p < 0.05$, **: $p < 0.01$ and ***: $p < 0.001$).

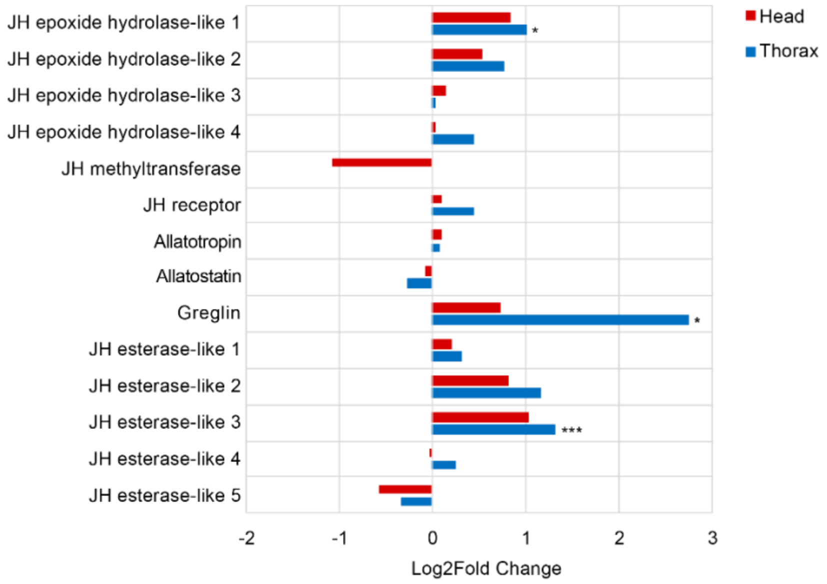

SUPPLEMENTARY TABLES

Supplementary Table 1: Nonparametric multivariate analysis of behavioural variables. Shown are *p* values from the nonparametric multivariate analyses using nparMD. The presented values are from the F-approximation for Dempster's ANOVA to allow for consistency in test statistics across all of our effects. Behavioural variables were grouped according to those that measured time and those that measured distance and analysed separately. Significant *p* values are shown in bold face. Degrees of freedom regarding the test for each grouping are given as $DF_{\#, \#}$.

Effect	*p* value	F-approximation
Time (seconds)		$DF_{7, 286}$
Density	< 0.001	90.362
Species	< 0.001	42.593
Density-Species	< 0.001	4.794
Distance (cm)		$DF_{2, 286}$
Density	< 0.001	95.830
Species	< 0.001	38.054
Density-Species	< 0.001	5.309

Supplementary Table 2: Statistical significance associated with nonzero gamma-distribution of two-step hurdle model analysis.

This table shows the *p* values associated with the nonzero gamma-distribution of the two-step hurdle models for each behavioural variable. All *p* values are adjusted for multiple comparisons using the Bonferonni correction. Significant values are put in bold.

Variable	Species				Density			
	piceifrons	*americana*	*cubense*	*nitens*	*piceifrons*	*americana*	*cubense*	*nitens*
Distance moved	<0.001	<0.001	<0.001	<0.001	<0.001	<0.001	<0.001	<0.001
Movement	<0.001	<0.001	<0.001	<0.001	<0.001	<0.001	<0.001	<0.001
Stimulus zone time	1.000	0.607	<0.001	<0.001	1.000	0.260	<0.001	1.000
Non-stimulus zone time	1.000	1.000	1.000	<0.001	<0.001	0.005	0.039	0.154
Neutral zone time	0.092	1.000	<0.001	<0.001	0.003	<0.001	0.017	1.000
Climbing time	1.000	1.000	0.224	<0.001	0.201	1.000	1.000	1.000
Rotation frequency	<0.001	<0.001	<0.001	<0.001	<0.001	<0.001	<0.001	<0.001
Stimulus wall time	1.000	0.215	0.101	<0.001	1.000	0.524	0.075	1.000
Distance to stimulus zone	1.000	1.000	1.000	<0.001	0.572	<0.001	1.000	1.000
Velocity	0.221	<0.001	1.000	0.015	1.000	<0.001	0.002	<0.001

Supplementary Table 3: Statistical significance associated with the logistic regression step of the two-step hurdle model analysis.

This table shows the *p* values associated with the logistic regression step of the two-step hurdle models for each behavioural variable. All *p* values are adjusted for multiple comparisons using the Bonferonni correction. Significant values are put in bold.

Variable	Species				Density			
	piceifrons	*americana*	*cubense*	*nitens*	*piceifrons*	*americana*	*cubense*	*nitens*
Distance moved	1.000	1.000	1.000	1.000	1.000	1.000	1.000	1.000
Movement	1.000	1.000	1.000	1.000	1.000	1.000	1.000	1.000
Stimulus zone time	1.000	1.000	1.000	0.929	<0.001	0.001	0.024	0.291
Non-stimulus zone time	0.653	0.020	0.051	0.129	1.000	1.000	1.000	1.000
Neutral zone time	1.000	1.000	1.000	0.002	0.073	0.192	1.000	1.000
Climbing time	1.000	0.073	0.139	0.027	1.000	1.000	1.000	1.000
Rotation frequency	1.000	0.138	1.000	0.002	1.000	1.000	0.842	0.590
Stimulus wall time	1.000	0.414	1.000	<0.001	<0.001	<0.001	<0.001	0.001
Distance to stimulus zone	1.000	1.000	1.000	0.001	0.046	0.024	0.192	0.368
Velocity	1.000	1.000	1.000	1.000	1.000	1.000	1.000	1.000

Supplementary Table 4: Statistical significance associated with three-way ANOVA for the pronotum length.
This table shows the *p* values associated with the three-way ANOVA testing the effect of sex, species and rearing condition on the linear measurement of the pronotum length. Significant *p* values are put in bold.

	Df	Sum Sq	Mean Sq	F value	Pr(>F)
Sex	1	1.1571	1.1571	932.479	$< 2*10^{-16}$
Species	3	0.6333	0.2111	170.114	$< 2*10^{-16}$
RearingCondition	1	0.0007	0.0007	0.573	0.45
Sex:Species	3	0.3550	0.1183	95.356	$< 2*10^{-16}$
Sex:RearingCondition	1	0.0602	0.0602	48.529	$3.19*10^{-11}$
Species:RearingCondition	3	0.0738	0.0246	19.812	$1.71*10^{-11}$
Sex:Species:RearingCondition	3	0.0017	0.0006	0.468	0.705
Residuals	237	0.2941	0.0012		

Supplementary Table 5: Statistical significance associated with three-way ANOVA for the F/C ratio.
This table shows the *p* values associated with the three-way ANOVA testing the effect of sex, species and rearing condition on the F/C morphometric ratio. Significant *p* values are put in bold.

	Df	Sum Sq	Mean Sq	F value	Pr (>F)
Sex	1	0.001	0.001	0.066	0.7979
Species	3	1.206	0.402	34.669	$< 2*10^{-16}$
RearingCondition	1	3.757	3.757	324.056	$< 2*10^{-16}$
Sex:Species	3	0.393	0.131	11.31	$5.84*10^{-7}$
Sex:RearingCondition	1	0.048	0.048	4.18	0.042
Species:RearingCondition	3	0.63	0.21	18.12	$1.28*10^{-10}$
Sex:Species:RearingCondition	3	0.124	0.041	3.579	0.0146
Residuals	237	2.748	0.012		

Supplementary Table 6: Transcriptome statistics for all eight transcriptomes.
Shown are the number of filtered contigs, the GC percentage, the contig N50, the number of filtered reads used for each assembly, and the Transcriptome Shotgun Assembly accession number for each of the eight transcriptomes generated during this study.

Species	Tissue	Total contigs	GC percentage	Contig N50	Reads
piceifrons	head	308,576	40.79	655	389,743,930
	thorax	282,538	41.27	627	386,113,748
americana	head	342,815	40.86	597	418,695,940
	thorax	305,904	41.17	622	424,852,038
cubense	head	374,276	41.14	578	456,654,000
	thorax	307,621	41.23	634	457,347,006
nitens	head	312,411	41.32	623	372,074,768
	thorax	278,171	41.6	674	405,833,792

Supplementary Table 7: Quality assessment for transcriptomes generated during this study. This table contains the results of various assessments of the quality of each final transcriptome, after all filtering steps were performed. The amount of total contigs, CG percentage and N50 were obtained with Trinitystats. BUSCO was used to calculate the percentage of single-copy orthologues from the lineage Insecta that were present in each transcriptome. The percentage of reads that mapped back to the transcriptome was obtained with bowtie2 and flagstat. The transrate score was a result of running Transrate on the final transcriptomes. Finally, the percentages of annotated genes and genes with a BLAST hit were obtained by Blast2GO.

	piceifrons		*americana*		*cubense*		*nitens*	
	Head	Thorax	Head	Thorax	Head	Thorax	Head	Thorax
Total contigs	308576	282538	342815	305904	374276	307621	312411	278171
GC percentage	40.79	41.27	40.86	41.17	41.14	41.23	41.32	41.6
Contig N50	655	627	597	622	578	634	623	674
Complete BUSCOs (%)	95.3	96.4	96.5	96.3	96.4	96.4	95.9	96.2
Single-copy BUSCOs (%)	79.6	80.1	81.5	80.5	80.1	82.3	80.3	79.9
Fragmented BUSCOs (%)	2.5	1.9	2.1	2.1	2.2	2	2.4	2.4
Reads mapped back (%)	88.47	86.76	89.08	85.2	87.89	84.12	89.25	86.82
Transrate score	0.3199	0.3126	0.3310	0.3242	0.2974	0.3108	0.3325	0.3158
Annotated (%)	7.4	7.7	6.8	7.3	6.7	7.4	7.7	8.4
Blast hit (%)	22.3	22.6	22	23.1	22.1	23.4	23.2	24.5

Supplementary Table 8: Contamination of transcriptomes of *piceifrons* and *americana*, generated during this study. The percentages of reads that mapped to different sources of contamination were obtained with FastQScreen. Sources of contamination were chosen by conducting a BLAST search earlier draft versions of the transcriptomes to potential contaminants. '% mapping' represents the percentage of reads that match the contamination source, while '% uniquely mapping' represents the percentage of reads that only maps to that particular contaminating source but not to any other of the analysed sources.

	piceifrons head		*piceifrons* thorax		*americana* head		*americana* thorax	
	% mapping	% uniquely mapping	% mapping	% uniquely mapping	% mapping	% uniquely mapping	% mapping	% uniquely mapping
Adapters	0.00025	0.00000	0.00021	0.00000	0.00019	0.00000	0.00017	0.00000
Bosea sp	0.06469	0.00117	0.05448	0.00078	0.06516	0.00102	0.05838	0.00088
Bradyrhizobium sp	0.77068	0.00744	0.72147	0.00644	0.79877	0.00665	0.71573	0.01205
Escherichia coli K12	0.00492	0.00013	0.00362	0.00016	0.00589	0.00011	0.00367	0.00017
Encephalitozoon romaleae	0.00230	0.00078	0.00212	0.00114	0.00184	0.00054	0.00365	0.00274
Gregarina niphandrodes	0.81315	0.00030	0.76047	0.00035	0.85253	0.00033	0.76549	0.00033
Homo sapiens (hg19)	3.44557	0.07187	3.22000	0.06686	3.45527	0.06477	3.02442	0.06339
Klebsiella pneumoniae	0.00476	0.00001	0.00342	0.00001	0.00581	0.00004	0.00347	0.00002
Locusta migratoria	42.83589	35.26244	38.36251	32.25639	41.94645	34.71305	37.77929	32.22948
Methylobacterium sp	0.08889	0.00367	0.08430	0.01102	0.09470	0.00527	0.09062	0.00986
PhiX	0.00075	0.00000	0.00075	0.00000	0.00135	0.00000	0.00074	0.00000
Propionibacterium acnes	0.00270	0.00025	0.00218	0.00012	0.00333	0.00007	0.00267	0.00040
Rhodopseudomonas sp	0.05821	0.00042	0.05242	0.00042	0.06555	0.00062	0.06009	0.00041
Sphingomonas sp	0.86414	0.02245	0.73486	0.02020	0.84395	0.04049	0.69599	0.02197
UniVec	0.00287	0.00003	0.00267	0.00003	0.00476	0.00003	0.00332	0.00004
Zootermopsis nevadensis	6.60033	0.37566	5.12609	0.24532	6.27598	0.34145	4.63494	0.19973
not mapping	57.83571		61.27663		58.56452		62.60714	

Supplementary Table 9: Contamination of transcriptomes of *cubense* and *nitens*, generated during this study. The percentages of reads that mapped to different sources of contamination were obtained with FastQScreen. Sources of contamination were chosen by conducting a BLAST search earlier draft versions of the transcriptomes to potential contaminants. '% mapping' represents the percentage of reads that match the contamination source, while '% uniquely mapping' represents the percentage of reads that only maps to that particular contaminating source but not to any other of the analysed sources.

	cubense head		*cubense* thorax		*nitens* head		*nitens* thorax	
	% mapping	% uniquely mapping	% mapping	% uniquely mapping	% mapping	% uniquely mapping	% mapping	% uniquely mapping
Adapters	0.00025	0.00000	0.00030	0.00000	0.00039	0.00000	0.00026	0.00000
Bosea sp	0.06576	0.00157	0.06032	0.00174	0.06886	0.00269	0.05585	0.00153
Bradyrhizobium sp	0.76958	0.00579	0.69142	0.01050	0.76752	0.01074	0.67466	0.00797
Escherichia coli K12	0.00478	0.00006	0.00385	0.00008	0.00599	0.00009	0.00482	0.00011
Encephalitozoon romaleae	0.00157	0.00041	0.00320	0.00254	0.00273	0.00121	0.44095	0.43957
Gregarina niphandrodes	0.81997	0.00026	0.74670	0.00031	0.74681	0.00080	0.66602	0.00052
Homo sapiens (hg19)	3.72328	0.07172	3.21871	0.07146	3.59714	0.08104	2.95039	0.06852
Klebsiella pneumoniae	0.00473	0.00003	0.00379	0.00002	0.00598	0.00004	0.00476	0.00001
Locusta migratoria	41.35465	33.90888	36.54207	30.83197	42.16382	35.20375	36.43013	31.12450
Methylobacterium sp	0.09394	0.00432	0.09253	0.01009	0.09719	0.00442	0.09040	0.01291
PhiX	0.00207	0.00000	0.00132	0.00000	0.00087	0.00000	0.00154	0.00000
Propionibacterium acnes	0.00261	0.00012	0.00441	0.00090	0.00384	0.00116	0.00264	0.00018
Rhodopseudomonas sp	0.06295	0.00047	0.05983	0.00052	0.06093	0.00058	0.05015	0.00042
Sphingomonas sp	0.88416	0.03998	0.73372	0.02419	0.85018	0.05534	0.66417	0.01312
UniVec	0.00435	0.00004	0.00384	0.00005	0.00299	0.00004	0.00333	0.00004
Zootermopsis nevadensis	6.26880	0.33708	4.61061	0.19930	5.74122	0.28359	4.31184	0.15946
not mapping	59.49569		63.98066		58.24665		63.17737	

Supplementary Table 10: Transposons present in transcriptomes generated during this study.
The presence of transposon sequences was calculated with RepeatFinder. Each value represents the percentage of nucleotides of the assembly that matches a particular group of transposons or other repeats.

			piceifrons		*americana*		*cubense*		*nitens*	
			Head	Thorax	Head	Thorax	Head	Thorax	Head	Thorax
Retroelements	SINE	Retroelements	11.74	11.65	11.74	11.71	11.97	11.69	11.25	11.25
		SINE	0.57	0.6	0.58	0.59	0.61	0.6	0.58	0.57
	LINE	Penelope	2.33	2.35	2.32	2.35	2.35	2.3	2.22	2.24
		LINE	9.57	9.48	9.63	9.56	9.71	9.5	9.15	9.12
		CRE/SLACS	0	0	0	0	0	0	0	0
		L2/CR1/Rex	3.48	3.3	3.51	3.38	3.53	3.43	3.33	3.15
		R1/LOA/Jockey	1.37	1.53	1.37	1.47	1.42	1.45	1.3	1.43
		R2/R4/NeSL	0.01	0.01	0.01	0.01	0.01	0.01	0.01	0.01
		RTE/Bov-B	1.85	1.75	1.86	1.78	1.83	1.76	1.74	1.7
	LTR	L1/CIN4	0	0	0	0	0	0	0	0
		LTR	1.59	1.57	1.53	1.57	1.64	1.59	1.52	1.56
		BEL/Pao	0.23	0.24	0.23	0.24	0.25	0.24	0.23	0.23
		Ty1/Copia	0.13	0.12	0.13	0.12	0.12	0.11	0.11	0.11
DNA-transposons		Gypsy/DIRS1	1.23	1.22	1.17	1.21	1.27	1.23	1.18	1.21
		DNA transposons	5.89	5.89	5.98	6.01	6.1	6.05	5.87	5.87
		Hobo-Activator	0.58	0.58	0.6	0.59	0.6	0.59	0.58	0.55
		Tc1-IS630-Pogo	3.79	3.81	3.86	3.92	3.97	3.97	3.8	3.88
		En-Spm	0	0	0	0	0	0	0	0
		MuDR-IS905	0	0	0	0	0	0	0	0
		PiggyBac	0.26	0.26	0.26	0.25	0.25	0.26	0.25	0.24
		Tourist/Harbinger	0.06	0.06	0.06	0.06	0.07	0.07	0.07	0.07
		Other (Mirage, P-element, Transib)	0.02	0.03	0.02	0.02	0.02	0.02	0.02	0.02
		Rolling-circles	0	0	0	0	0	0	0	0
		Unclassified:	0.51	0.49	0.53	0.51	0.53	0.51	0.48	0.48

Supplementary Table 11: Comparison of read mapping to own transcriptome and *piceifrons* transcriptome.
Filtered reads were either mapped to their own transcriptome or to the representative transcriptome of *piceifrons*, using bowtie2. The percentage of reads mapping to the transcriptome was calculated by Flagstat. Differentially expressed genes were identically obtained with DEseq2 and edgeR. Any gene that exhibited an absolute Log_2FoldChange larger than 1 and an adjusted *p* value smaller than 0.05 for any of the two programs was considered a differentially expressed gene.

		To own transcriptome		To *piceifrons* transcriptome	
		% reads mapped	DE genes	% reads mapped	DE genes
piceifrons	Head	88.47	1317	88.47	1317
	Thorax	86.76	3138	86.76	3138
americana	Head	89.08	1127	86.87	1162
	Thorax	85.2	1033	85	1015
cubense	Head	87.89	85	86.38	47
	Thorax	84.12	306	84.1	259
nitens	Head	89.25	175	84.17	97
	Thorax	86.82	14	80.63	0

Supplementary Table 12: Overview of the nine hexamerin-like proteins found in four *Schistocerca* species. Genbank accession numbers were given for the hexamerin-like proteins of all four species. We named six hexamerin-like proteins based on their homology with *L. migratoria* hexamerin-like proteins, and added hexamerin-like protein 7-9 as their *L. migratoria* orthologue were never given a serial name. Additionally, for each gene the closest orthologue in *piceifrons* was given in addition to the percentage of identities at the nucleotide level (% identity). For each gene, the closest orthologue in *L. migratoria* and its GenBank accession number – if available – was given. Last, the differential expression for each gene in all three studied locust species (*piceifrons*, *L. migratoria*, *S. gregaria*) was listed.

Gene name	Closest orthologue in *piceifrons*	% identity	Orthologue in *L. migratoria*	Membership 'gregarious behaviour' module	Differential expression *piceifrons*	Differential expression *L. migratoria*	Differential expression *S. gregaria*
Hexamerin-like protein 7	Hexamerin-like protein 6	64.28	LMC_001181 (Kang et al., 2004)	head + thorax	Upregulated	Upregulated (Kang et al., 2004)	
Hexamerin-like protein 5	Hexamerin-like protein 8	60.75	hexamerin-like protein 5	thorax	Upregulated		
Hexamerin-like protein 4	Hexamerin-like protein 6	56.16	hexamerin-like protein 4		Upregulated	Upregulated (Yang et al., 2019)	
Hexamerin-like protein 6	Hexamerin-like protein 2a	78.66	hexamerin-like protein 6	head + thorax	Upregulated	Upregulated (Kang et al., 2004)	
Hexamerin-like protein 8	Hexamerin-like protein 5	60.75	juvenile hormone binding protein		Upregulated		
Hexamerin-like protein 2a	Hexamerin-like protein 2b	90	hexamerin-like 2/3	head + thorax	Upregulated	Upregulated (Kang et al., 2004)	Downregulated (Badisco et al.)
Hexamerin-like protein 2b	Hexamerin-like protein 2a	90	hexamerin-like 2/3	thorax	Upregulated		
Hexamerin-like protein 1	Hexamerin-like protein 9	73.11	hexamerin-like protein 1		Downregulated	Upregulated (Yang et al., 2019)	
Hexamerin-like protein 9	Hexamerin-like protein 1	73.11	hexamerin, partial cds		Downregulated	Upregulated (Yang et al., 2019)	

Supplementary Table 13: Genbank accession numbers for Hexamerin-like proteins in four *Schistocerca* species

Gene name	Genbank accession number			
	piceifrons	*americana*	*cubense*	*nitens*
Hexamerin-like protein 7	MT877466	MT877467	MT877465	MT877464
Hexamerin-like protein 5	MT877479	MT877478	MT877477	MT877476
Hexamerin-like protein 4	MT877471	MT877469	MT877470	MT877468
Hexamerin-like protein 6	MT877487	MT877486	MT877485	MT877484
Hexamerin-like protein 8	MT877491	MT877490	MT877489	MT877488
Hexamerin-like protein 2a	MT877475	MT877474	MT877473	MT877472
Hexamerin-like protein 2b	MT877483	MT877481	MT877480	MT877482
Hexamerin-like protein 1	MT877460	MT877462	MT877461	MT877463
Hexamerin-like protein 9	MT877459	MT877458	MT877456	MT877457

SUPPLEMENTARY DATA FILES AND DESCRIPTIONS PROVIDED VIA THE DIGITAL REPOSITORY DRYAD

Files can be found here: https://doi.org/10.5061/dryad.dz08kprwz

Supplementary Data File 1: Differentially expressed genes for the head tissue of *piceifrons*, and their annotation.
This table lists all differentially expressed genes for the head tissue of *piceifrons*, discovered by either DEseq2 or edgeR, and their annotation by Blast2GO. For both DEseq2 and edgeR, the Log_2FoldChange (Log_2FC) and adjusted *p* value (padj) were given for each contig. Additionally, gene annotation was obtained with Blast2GO, and the following outputs were given: the gene description, based on BLAST hits (Gene description), contig length (Length), the Expect value associated with the best BLAST hit (E value), the mean similarity at the nucleotide level to its BLAST hits (Similarity), the Gene Ontology IDs (GO IDs) and Gene Ontology names (GO names), and finally the annotation confidence level, for which 'interpro' refers to sequences with a hit to the interpro database, 'blasted' refers to sequences with at least one BLAST hit to the arthropod nr database, 'mapped' was given to sequences for which gene ontology information could be retrieved from its BLAST hit(s), and 'annotated' was only given to sequences for which the mapped GO terms also scored above the default thresholds for annotation.

Supplementary Data File 2: Differentially expressed genes for the thorax tissue of *piceifrons*, and their annotation.
This table lists all differentially expressed genes for the thorax tissue of *piceifrons*, discovered by either DEseq2 or edgeR, and their annotation by Blast2GO. For both DEseq2 and edgeR, the Log_2FoldChange (Log_2FC) and adjusted *p* value (padj) were given for each contig. Additionally, gene annotation was obtained with Blast2GO, and the following outputs were given: the gene description, based on BLAST hits (Gene description), contig length (Length), the Expect value associated with the best BLAST hit (E value), the mean similarity at the nucleotide level to its BLAST hits (Similarity), the Gene Ontology IDs (GO IDs) and Gene Ontology names (GO names), and finally the annotation confidence level, for which 'interpro' refers to sequences with a hit to the interpro database, 'blasted' refers to sequences with at least one BLAST hit to the arthropod nr database, 'mapped' was given to sequences for which gene ontology information could be retrieved from its BLAST hit(s), and 'annotated' was only given to sequences for which the mapped GO terms also scored above the default thresholds for annotation.

Supplementary Data File 3: Differentially expressed genes for the head tissue of *americana*, and their annotation.
This table lists all differentially expressed genes for the head tissue of *americana*, discovered by either DEseq2 or edgeR, and their annotation by Blast2GO. For both DEseq2 and edgeR, the Log_2FoldChange (Log_2FC) and adjusted *p* value (padj) were given for each contig. Additionally, gene annotation was obtained with Blast2GO, and the following outputs were given: the gene description, based on BLAST hits (Gene

description), contig length (Length), the Expect value associated with the best BLAST hit (E value), the mean similarity at the nucleotide level to its BLAST hits (Similarity), the Gene Ontology IDs (GO IDs) and Gene Ontology names (GO names), and finally the annotation confidence level, for which ‘interpro’ refers to sequences with a hit to the interpro database, ‘blasted’ refers to sequences with at least one BLAST hit to the arthropod nr database, ‘mapped’ was given to sequences for which gene ontology information could be retrieved from its BLAST hit(s), and ‘annotated’ was only given to sequences for which the mapped GO terms also scored above the default thresholds for annotation.

Supplementary Data File 4: Differentially expressed genes for the thorax tissue of *americana*, and their annotation.
This table lists all differentially expressed genes for the thorax tissue of *americana*, discovered by either DEseq2 or edgeR, and their annotation by Blast2GO. For both DEseq2 and edgeR, the Log_2FoldChange (Log_2FC) and adjusted *p* value (padj) were given for each contig. Additionally, gene annotation was obtained with Blast2GO, and the following outputs were given: the gene description, based on BLAST hits (Gene description), contig length (Length), the Expect value associated with the best BLAST hit (E value), the mean similarity at the nucleotide level to its BLAST hits (Similarity), the Gene Ontology IDs (GO IDs) and Gene Ontology names (GO names), and finally the annotation confidence level, for which ‘interpro’ refers to sequences with a hit to the interpro database, ‘blasted’ refers to sequences with at least one BLAST hit to the arthropod nr database, ‘mapped’ was given to sequences for which gene ontology information could be retrieved from its BLAST hit(s), and ‘annotated’ was only given to sequences for which the mapped GO terms also scored above the default thresholds for annotation.

Supplementary Data File 5: Differentially expressed genes for the head tissue of *cubense*, and their annotation.
This table lists all differentially expressed genes for the head tissue of *cubense*, discovered by either DEseq2 or edgeR, and their annotation by Blast2GO. For both DEseq2 and edgeR, the Log_2FoldChange (Log_2FC) and adjusted *p* value (padj) were given for each contig. Additionally, gene annotation was obtained with Blast2GO, and the following outputs were given: the gene description, based on BLAST hits (Gene description), contig length (Length), the Expect value associated with the best BLAST hit (E value), the mean similarity at the nucleotide level to its BLAST hits (Similarity), the Gene Ontology IDs (GO IDs) and Gene Ontology names (GO names), and finally the annotation confidence level, for which ‘interpro’ refers to sequences with a hit to the interpro database, ‘blasted’ refers to sequences with at least one BLAST hit to the arthropod nr database, ‘mapped’ was given to sequences for which gene ontology information could be retrieved from its BLAST hit(s), and ‘annotated’ was only given to sequences for which the mapped GO terms also scored above the default thresholds for annotation.

Supplementary Data File 6: Differentially expressed genes for the thorax tissue of *cubense*, and their annotation.

This table lists all differentially expressed genes for the thorax tissue of *cubense*, discovered by either DEseq2 or edgeR, and their annotation by Blast2GO. For both DEseq2 and edgeR, the Log_2FoldChange (Log_2FC) and adjusted *p* value (padj) were given for each contig. Additionally, gene annotation was obtained with Blast2GO, and the following outputs were given: the gene description, based on BLAST hits (Gene description), contig length (Length), the Expect value associated with the best BLAST hit (E value), the mean similarity at the nucleotide level to its BLAST hits (Similarity), the Gene Ontology IDs (GO IDs) and Gene Ontology names (GO names), and finally the annotation confidence level, for which 'interpro' refers to sequences with a hit to the interpro database, 'blasted' refers to sequences with at least one BLAST hit to the arthropod nr database, 'mapped' was given to sequences for which gene ontology information could be retrieved from its BLAST hit(s), and 'annotated' was only given to sequences for which the mapped GO terms also scored above the default thresholds for annotation.

Supplementary Data File 7: Differentially expressed genes for the head tissue of *nitens*, and their annotation.
This table lists all differentially expressed genes for the head tissue of *nitens*, discovered by either DEseq2 or edgeR, and their annotation by Blast2GO. For both DEseq2 and edgeR, the Log_2FoldChange (Log_2FC) and adjusted *p* value (padj) were given for each contig. Additionally, gene annotation was obtained with Blast2GO, and the following outputs were given: the gene description, based on BLAST hits (Gene description), contig length (Length), the Expect value associated with the best BLAST hit (E value), the mean similarity at the nucleotide level to its BLAST hits (Similarity), the Gene Ontology IDs (GO IDs) and Gene Ontology names (GO names), and finally the annotation confidence level, for which 'interpro' refers to sequences with a hit to the interpro database, 'blasted' refers to sequences with at least one BLAST hit to the arthropod nr database, 'mapped' was given to sequences for which gene ontology information could be retrieved from its BLAST hit(s), and 'annotated' was only given to sequences for which the mapped GO terms also scored above the default thresholds for annotation.

Supplementary Data File 8: Differentially expressed genes for the thorax tissue of *nitens*, and their annotation.
This table lists all differentially expressed genes for the thorax tissue of *nitens*, discovered by either DEseq2 or edgeR, and their annotation by Blast2GO. For both DEseq2 and edgeR, the Log_2FoldChange (Log_2FC) and adjusted *p* value (padj) were given for each contig. Additionally, gene annotation was obtained with Blast2GO, and the following outputs were given: the gene description, based on BLAST hits (Gene description), contig length (Length), the Expect value associated with the best BLAST hit (E value), the mean similarity at the nucleotide level to its BLAST hits (Similarity), the Gene Ontology IDs (GO IDs) and Gene Ontology names (GO names), and finally the annotation confidence level, for which 'interpro' refers to sequences with a hit to the interpro database, 'blasted' refers to sequences with at least one BLAST hit to the arthropod nr database, 'mapped' was given to sequences for which gene ontology information could be retrieved from its BLAST hit(s), and 'annotated' was only given to

sequences for which the mapped GO terms also scored above the default thresholds for annotation.

Supplementary Data File 9: Differentially expressed genes for all head tissues using the *piceifrons* transcriptome as reference, and their annotation.
This table lists differentially expressed genes for the head tissues of *piceifrons*, *americana*, *cubense* and *nitens*, using the *piceifrons* transcriptome as reference. After aligning all filtered reads for the different species to the *piceifrons* head transcriptome, differentially expressed genes were calculated with both DEseq2 and EdgeR for each species separately. Any gene that is differentially expressed in at least one species, was included in the table. For both DEseq2 and edgeR, the Log_2FoldChange (Log_2FC) and adjusted *p* value (padj) were listed. Additionally, gene annotation was obtained with Blast2GO, and the following outputs were given: the gene description, based on BLAST hits (Gene description), contig length (Length), the Expect value associated with the best BLAST hit (E value), the mean similarity at the nucleotide level to its BLAST hits (Similarity), the Gene Ontology IDs (GO IDs) and Gene Ontology names (GO names), and finally the annotation confidence level, for which 'interpro' refers to sequences with a hit to the interpro database, 'blasted' refers to sequences with at least one BLAST hit to the arthropod nr database, 'mapped' was given to sequences for which gene ontology information could be retrieved from its BLAST hit(s), and 'annotated' was only given to sequences for which the mapped GO terms also scored above the default thresholds for annotation.

Supplementary Data File 10: Differentially expressed genes for all thorax tissues using the *piceifrons* transcriptome as reference, and their annotation.
This table lists differentially expressed genes for the thorax tissues of *piceifrons*, *americana*, *cubense* and *nitens*, using the *piceifrons* transcriptome as reference. After aligning all filtered reads for the different species to the *piceifrons* thorax transcriptome, differentially expressed genes were calculated with both DEseq2 and EdgeR for each species separately. Any gene that is differentially expressed in at least one species, was included in the table. For both DEseq2 and edgeR, the Log_2FoldChange (Log_2FC) and adjusted *p* value (padj) were listed. Additionally, gene annotation was obtained with Blast2GO, and the following outputs were given: the gene description, based on BLAST hits (Gene description), contig length (Length), the Expect value associated with the best BLAST hit (E value), the mean similarity at the nucleotide level to its BLAST hits (Similarity), the Gene Ontology IDs (GO IDs) and Gene Ontology names (GO names), and finally the annotation confidence level, for which 'interpro' refers to sequences with a hit to the interpro database, 'blasted' refers to sequences with at least one BLAST hit to the arthropod nr database, 'mapped' was given to sequences for which gene ontology information could be retrieved from its BLAST hit(s), and 'annotated' was only given to sequences for which the mapped GO terms also scored above the default thresholds for annotation.

Supplementary Data File 11: Enrichment of Gene Ontology terms of differentially expressed genes, for each species and tissue combination.

Fisher exact tests were performed in Blast2GO by comparing the Gene Ontology terms in the set of differentially expressed genes to the ones in the whole transcriptome. Results obtained for the four different species and two different tissues were all pooled together in a single table. The first three columns contain the full name (GO name), ID (GO ID) and category (GO category) of each GO term. For each species and tissue combination, the table contains the False Discovery Rate associated with a certain GO term (FDR), the amount present in the differentially expressed genes (# in DE genes) and in the whole transcriptome (# overall), and last the percentage of genes that were annotated with that specific annotation term within the set of all DE genes (% in DE genes) or within the full transcriptome (% overall).

Supplementary Data File 12: WGCNA module membership for each gene in the head tissue.
Modules of co-expressed genes for the head tissue were obtained with WGCNA. For each contig, the module for which it obtained the highest membership value was listed in the column 'Module'. As an estimate of the importance of the particular contig in explaining the variation of phenotypic traits, a gene significance (GS) value and an associated *p* value was calculated for each tested behavioural trait. Additionally, the module membership (MM) of each gene was calculated for each module, in addition to the associated *p* value. Finally, the table contains the gene annotation of Blast2GO for each gene. The following outputs were given: the gene description, based on BLAST hits (Gene description), contig length (Length), the Expect value associated with the best BLAST hit (E value), the mean similarity at the nucleotide level to its BLAST hits (Similarity), the Gene Ontology IDs (GO IDs) and Gene Ontology names (GO names), and finally the annotation confidence level, for which 'interpro' refers to sequences with a hit to the interpro database, 'blasted' refers to sequences with at least one BLAST hit to the arthropod nr database, 'mapped' was given to sequences for which gene ontology information could be retrieved from its BLAST hit(s), and 'annotated' was only given to sequences for which the mapped GO terms also scored above the default thresholds for annotation.

Supplementary Data File 13: WGCNA module membership for each gene in the thorax tissue.
Modules of co-expressed genes for the thorax tissue were obtained with WGCNA. For each contig, the module for which it obtained the highest membership value was listed in the column 'Module'. As an estimate of the importance of the particular contig in explaining the variation of phenotypic traits, a gene significance (GS) value and an associated *p* value was calculated for each tested behavioural trait. Additionally, the module membership (MM) of each gene was calculated for each module, in addition to the associated *p* value. Finally, the table contains the gene annotation of Blast2GO for each gene. The following outputs were given: the gene description, based on BLAST hits (Gene description), contig length (Length), the Expect value associated with the best BLAST hit (E value), the mean similarity at the nucleotide level to its BLAST hits (Similarity), the Gene Ontology IDs (GO IDs) and Gene Ontology names (GO names), and finally the annotation confidence level, for which 'interpro' refers to sequences with a hit to the interpro database, 'blasted' refers to sequences with at least one BLAST hit to

the arthropod nr database, ‘mapped’ was given to sequences for which gene ontology information could be retrieved from its BLAST hit(s), and ‘annotated’ was only given to sequences for which the mapped GO terms also scored above the default thresholds for annotation.

Supplementary Data File 14: Archive (zip) file containing: (a) Raw data from behavioural assays for all four species of *Schistocerca* (Behaviour_dataset.csv); (b) RMarkdown file used for analysing behavioural reaction norm data (Rmarkdown_behaviour.Rmd); (c) Html file describing codes used for analysing behavioural reaction norm data (Rmarkdown_behaviour.html)

Supplementary Data File 15: Archive (zip) file containing: (a) Raw colour data (jpg images and coordinates) for all four species of *Schistocerca* for running the program patternize (folder: Raw_colour_data_and_coordinates); (b) RMarkdown file used for running patternize to analyse colour reaction norm data (Rmarkdown_colour.Rmd); (c) Html file describing codes used for running patternize to analyse colour reaction norm data (Rmarkdown_colour.html)

Supplementary Data File 16: Archive (zip) file containing: (a) Raw data from morphological measurements for all four species of *Schistocerca* (Morphology_dataset.csv); (b) RMarkdown file used for analysing morphological reaction norm data (Rmarkdown_morphology.Rmd); (c) Html file describing codes used for analysing morphological reaction norm data (Rmarkdown_morphology.html)

Supplementary Data File 17: Archive (zip) file containing: (1) Text file describing R scripts for running edgeR and DESeq2 using SARTools (Code_DE_analyses.txt) [raw reads used for these analyses are included in Supplementary Data File 18]

Supplementary Data File 18: Archive (zip) file containing: (a) Raw data (raw count data and phenotypic trait data) for all four species of *Schistocerca* for running the program WGCNA (folder: Raw_data_for_DE_WGCNA); (b) RMarkdown file used for running WGCNA (Rmarkdown_WGCNA.Rmd); (c) Html file describing codes used for running WGCNA (Rmarkdown_WGCNA.html)

Supplementary Data File 19: Archive (zip) file containing: (a) FASTA file containing the alignment used for building the phylogeny of hexamerins (hexamerin.fasta); (b) Phylip file containing the alignment used for building the phylogeny of hexamerins (hexamerin.phy); (c) Newick file containing the maximum likelihood tree from the phylogenetic analysis of hexamerins (hexamerin.tre)

SUPPLEMENTARY REFERENCES

1 Gotham, S. & Song, H. Non-swarming grasshoppers exhibit density-dependent phenotypic plasticity reminiscent of swarming locusts. *J. Insect Physiol.* **59**, 1151-1159 (2013).

2 Kilpatrick, S. K. *et al.* Revealing hidden density-dependent phenotypic plasticity in sedentary grasshoppers in the genus *Schistocerca* Stål (Orthoptera: Acrididae: Cyrtacanthacridinae). *J. Insect Physiol.* **118**, 103937 (2019).

3 Roessingh, P., Simpson, S. J. & James, S. Analysis of phase-related changes in behaviour of desert locust nymphs. *Proc. R. Soc. B Biol. Sci.* **252**, 43-49 (1993).

4 Cullen, D. A., Sword, G. A. & Simpson, S. J. Optimizing multivariate behavioural syndrome models in locusts using automated video tracking. *Anim. Behav.* **84**, 771-784 (2012).

5 nparMD: Nonparametric Analysis of Multivariate Data in Factorial Designs. R package version 0.1.0. (https://CRAN.R-project.org/package=nparMD, 2018).

6 Hypervolume: high dimensional geometry and set operations using Kernel density estimation, support vector machines, and convex hulls. R package version 2.0.11. (https://CRAN.R-project.org/package=hypervolume, 2018).

7 R: A language and environment for statistical computing (R Foundation for Statistical Computing, Vienna, Austria, http://www.R-project.org/, 2017).

8 Van Belleghem, S. M. *et al.* patternize: an R package for quantifying colour pattern variation. *Methods in Ecology and Evolution* **9**, 390-398 (2018).

9 Schneider, C. A., Rasband, W. S. & Eliceiri, K. W. NIH Image to ImageJ: 25 years of image analysis. *Nature Methods* **9**, 671-675 (2012).

10 Dirsh, V. M. Morphometrical studies on phases of the desert locust. *Anti-Locust Bulletin* **16**, 1-34 (1953).

11 Uvarov, B. P. *Grasshoppers and Locusts, vol. 1*. Vol. 1 (Cambridge University Press, 1966).

12 Imbeaud, S. *et al.* Towards standardization of RNA quality assessment using user-independent classifiers of microcapillary electrophoresis traces. *Nucleic Acids Res.* **33**, e56-e56 (2005).

13 Escobar, M. D. & Hunt, J. L. A cost-effective RNA extraction technique from animal cells and tissue using silica columns. *J. Biol. Meth.* **4**, e72 (2017).

14 Winnebeck, E. C., Millar, C. D. & Warman, G. R. Why does insect RNA look degraded? *J. Insect Sci.* **10**, 159 (2010).

15 Macharia, R. W., Ombura, F. L. & Aroko, E. O. Insects' RNA profiling reveals absence of "hidden break" in 28S ribosomal RNA molecule of onion thrips, Thrips tabaci. *J. Nucleic Acids* **2015**, 965294 (2015).

16 Fabrick, J. A. & Hull, J. J. Assessing integrity of insect RNA 1-12 (Agilent Technologies Inc. , Application Note, 2017).

17 Afgan, E. *et al.* The Galaxy platform for accessible, reproducible and collaborative biomedical analyses: 2018 update. *Nucleic Acids Res.* **46**, W537-W544 (2018).

18 Blankenberg, D. *et al.* Manipulation of FASTQ data with Galaxy. *Bioinformatics* **26**, 1783-1785 (2010).

19 Bolger, A. M., Lohse, M. & Usadel, B. Trimmomatic: a flexible trimmer for Illumina sequence data. *Bioinformatics* **30**, 2114-2120 (2014).
20 Wingett, S. W. & Andrews, S. FastQ Screen: A tool for multi-genome mapping and quality control. *F1000Res.* **7**, 1338 (2018).
21 Grabherr, M. G. *et al.* Full-length transcriptome assembly from RNA-Seq data without a reference genome. *Nat. Biotechnol.* **29**, 644-652 (2011).
22 Li, W. & Godzik, A. Cd-hit: a fast program for clustering and comparing large sets of protein or nucleotide sequences. *Bioinformatics* **22**, 1658-1659 (2006).
23 Fu, L., Niu, B., Zhu, Z., Wu, S. & Li, W. CD-HIT: accelerated for clustering the next-generation sequencing data. *Bioinformatics* **28**, 3150-3152 (2012).
24 Smith-Unna, R., Boursnell, C., Patro, R., Hibberd, J. M. & Kelly, S. TransRate: reference-free quality assessment of de novo transcriptome assemblies. *Genome Res.* **26**, 1134-1144 (2016).
25 Simão, F. A., Waterhouse, R. M., Ioannidis, P., Kriventseva, E. V. & Zdobnov, E. M. BUSCO: assessing genome assembly and annotation completeness with single-copy orthologs. *Bioinformatics* **31**, 3210-3212 (2015).
26 Langmead, B. & Salzberg, S. L. Fast gapped-read alignment with Bowtie 2. *Nat. Meth.* **9**, 357-359 (2012).
27 Langmead, B., Trapnell, C., Pop, M. & Salzberg, S. L. Ultrafast and memory-efficient alignment of short DNA sequences to the human genome. *Genome Biol.* **10**, R25 (2009).
28 Li, H. Improving SNP discovery by base alignment quality. *Bioinformatics* **27**, 1157-1158 (2011).
29 Li, H. *et al.* The sequence alignment/map format and SAMtools. *Bioinformatics* **25**, 2078-2079 (2009).
30 Li, H. A statistical framework for SNP calling, mutation discovery, association mapping and population genetical parameter estimation from sequencing data. *Bioinformatics* **27**, 2987-2993 (2011).
31 Conesa, A. *et al.* Blast2GO: a universal tool for annotation, visualization and analysis in functional genomics research. *Bioinformatics* **21**, 3674-3676 (2005).
32 Quevillon, E. *et al.* InterProScan: protein domains identifier. *Nucleic Acids Res.* **33**, W116-W120 (2005).
33 Jones, P. *et al.* InterProScan 5: genome-scale protein function classification. *Bioinformatics* **30**, 1236-1240 (2014).
34 RepeatMasker Open-4.0. (http://www.repeatmasker.org, 2013).
35 Varet, H., Brillet-Guéguen, L., Coppée, J.-Y. & Dillies, M.-A. SARTools: a DESeq2-and edgeR-based R pipeline for comprehensive differential analysis of RNA-Seq data. *PLoS One* **11**, e0157022 (2016).
36 Robinson, M. D., McCarthy, D. J. & Smyth, G. K. edgeR: a Bioconductor package for differential expression analysis of digital gene expression data. *Bioinformatics* **26**, 139-140 (2010).
37 Love, M. I., Huber, W. & Anders, S. Moderated estimation of fold change and dispersion for RNA-seq data with DESeq2. *Genome Biol.* **15**, 550 (2014).
38 Wang, M., Zhao, Y. & Zhang, B. Efficient test and visualization of multi-set intersections. *Sci. Rep.* **5**, 16923 (2015).

39 Langfelder, P. & Horvath, S. WGCNA: an R package for weighted correlation network analysis. *BMC Bioinformatics* **9**, 559 (2008).
40 Zhang, B. & Horvath, S. A general framework for weighted gene co-expression network analysis. *Stat. Appl. Genet. Mol. Biol.* **4**, 17 (2005).
41 Langfelder, P., Zhang, B. & Horvath, S. Defining clusters from a hierarchical cluster tree: the Dynamic Tree Cut package for R. *Bioinformatics* **24**, 719-720 (2007).
42 Hu, Z. *et al.* VisANT 4.0: Integrative network platform to connect genes, drugs, diseases and therapies. *Nucleic Acids Res.* **41**, W225-W231 (2013).
43 Al-Shahrour, F., Díaz-Uriarte, R. & Dopazo, J. FatiGO: a web tool for finding significant associations of Gene Ontology terms with groups of genes. *Bioinformatics* **20**, 578-580 (2004).
44 Kang, L. *et al.* The analysis of large-scale gene expression correlated to the phase changes of the migratory locust. *Proc. Natl. Acad. Sci. U. S. A.* **101**, 17611-17615 (2004).
45 Yang, P., Hou, L., Wang, X. & Kang, L. Core transcriptional signatures of phase change in the migratory locust. *Protein Cell*, 1-19 (2019).
46 Edgar, R. C. MUSCLE: multiple sequence alignment with high accuracy and high throughput. *Nucleic Acids Res.* **32**, 1792-1797 (2004).
47 Stamatakis, A. RAxML version 8: a tool for phylogenetic analysis and post-analysis of large phylogenies. *Bioinformatics* **30**, 1312-1313 (2014).
48 The CIPRES Portals. CIPRES. (http://www.phylo.org/sub_sections/portal, 2011).
